# Production of indigo by recombinant bacteria

**DOI:** 10.1186/s40643-023-00626-7

**Published:** 2023-03-13

**Authors:** Julia A. Linke, Andrea Rayat, John M. Ward

**Affiliations:** 1 Chemical Engineering Department, University College London (UCL), Torrington Place, London, WC1E 7JE UK; 2 Division of Medicine, University College London (UCL), 5 University Street, London, WC1E 6JF UK; 3 Biochemical Engineering Department, University College London (UCL), Gower St., London, WC1E 6BT UK

**Keywords:** Bioprocess, Biocatalysis, Commercialization, Heterologous DNA expression, Recombinant biology, Scale up, Sustainability, Vat dye

## Abstract

Indigo is an economically important dye, especially for the textile industry and the dyeing of denim fabrics for jeans and garments. Around 80,000 tonnes of indigo are chemically produced each year with the use of non-renewable petrochemicals and the use and generation of toxic compounds. As many microorganisms and their enzymes are able to synthesise indigo after the expression of specific oxygenases and hydroxylases, microbial fermentation could offer a more sustainable and environmentally friendly manufacturing platform. Although multiple small-scale studies have been performed, several existing research gaps still hinder the effective translation of these biochemical approaches. No article has evaluated the feasibility and relevance of the current understanding and development of indigo biocatalysis for real-life industrial applications. There is no record of either established or practically tested large-scale bioprocess for the biosynthesis of indigo. To address this, upstream and downstream processing considerations were carried out for indigo biosynthesis. 5 classes of potential biocatalysts were identified, and 2 possible bioprocess flowsheets were designed that facilitate generating either a pre-reduced dye solution or a dry powder product. Furthermore, considering the publicly available data on the development of relevant technology and common bioprocess facilities, possible platform and process values were estimated, including titre, DSP yield, potential plant capacities, fermenter size and batch schedule. This allowed us to project the realistic annual output of a potential indigo biosynthesis platform as 540 tonnes. This was interpreted as an industrially relevant quantity, sufficient to provide an annual dye supply to a single industrial-size denim dyeing plant. The conducted sensitivity analysis showed that this anticipated output is most sensitive to changes in the reaction titer, which can bring a 27.8% increase or a 94.4% drop. Thus, although such a biological platform would require careful consideration, fine-tuning and optimization before real-life implementation, the recombinant indigo biosynthesis was found as already attractive for business exploitation for both, luxury segment customers and mass-producers of denim garments.

**Graphical Abstract:**

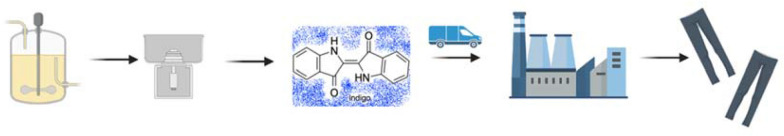

**Supplementary Information:**

The online version contains supplementary material available at 10.1186/s40643-023-00626-7.

## Introduction

Since ancient times colours and pigments were used by civilisations to record history in drawings, manuscripts and paintings (St Clair 2016). The indigo dye was among the first blue pigments to gain widespread use, especially for textile dyeing (Clark et al. 1993). Indigo still continues to be an economically important substance, with uses in many sectors. Examples include bioelectronic research for organic semi-conductors (Salzillo et al. 2018) or the medical fields, where a derivative, indigo carmine, may be used during surgeries (Craik et al. 2009). However, 95% of the globally produced dye is used solely by the textile industry. It is the leading dye for cotton denim fabric production and is used to dye jeans and over 4 billion denim garments each year (Schimper et al. 2011). With such remarkable market demand, the annual indigo production is estimated to be 70–80,000 tons (Wolf 2011; Garfield 2002; Paul et al. 2021).

For centuries indigo was extracted from plants via sophisticated processes, which were replaced by synthetic platforms during the nineteenth century chemical revolution (Balfour-Paul 2011). The current platforms use cheap, unrenewable petrochemical substrates and toxic chemicals, causing serious environmental damage. There is urgent need for more sustainable manufacture and several alternative approaches have been investigated, including semi-solutions, such as aniline-free synthesis or pre-reduced indigo (Scott 2015). However, environmental pollution would be better addressed with fully altered platforms, such as the use of microbial fermentation and recombinant biotechnology. These offer mild reaction conditions without toxic chemicals, use renewable sources and have been investigated on and off since the 1980s (Ensley et al. 1983; Murdock et al. 1993; Berry et al. 2002Hsu et al. 2018). The textile industry is resource-intensive and cost-sensitive, and commercialization of any bioprocess remained difficult due to the enormous cost-effectiveness of chemical synthesis (Toprak and Anis 2017).

The feasibility of recombinant indigo biosynthesis has been impeded by practical challenges of low yields, high cost of materials and of resources required for research toward optimization. For example, since the discovery of using recombinant microbes for indigo biosynthesis, 10 years passed until the development of a metabolic pathway from glucose (Murdock et al. 1993). Until today, no large-scale indigo biosynthesis has been established (Fabara and Fraaije 2020), although new driving factors have emerged, that may support changing that. The proceeding globalization, technological innovation and increasing pollution awareness result in rising taxation for the use and disposal of chemicals and a shift in customers’ preferences toward sustainable goods (Fernando et al. 2016). Bioengineering tools have also advanced exponentially, such as DNA sequencing, synthesis and biocatalysis (Khan et al. 2016). As a result, dye biosynthesis has been revisited by an increasing number of parties, including start-ups, such as PILI or Colorifix (www.pili.bio; www.colorifix.com). Still, the viability of these ventures relies on achieving certain cost-effectiveness and yields on top of the sustainability advantages. The aim of this study is to support the optimization and design of innovative, more sustainable indigo production platforms, which are in present times enhanced by the worldwide shift toward sustainability and greener manufacturing (Dornfeld 2014; Deloitte 2021).

Many wild-type and engineered bacteria have been identified to produce indigo via oxidation of its immediate indigo precursor, indole—Ma et al. (2018) published a table of all investigated enzymes and the hosts used with corresponding yield and a short description of each system. Fabara and Fraaije (2020) expanded this list and described in more detail the key catalysts, mainly different di- and monooxygenases, including aspects such as cofactors used. However, most studies did not expand outside the laboratory scale, except for Han et al. (2011), who investigated pilot-scale batch fermentation volume of 3000 L, and a 5 L continuous culture. Moreover, the scale-up of indigo biosynthesis and its economic feasibility have not been considered in the literature in much detail.

This study aims to expand the previous evaluations and consider which pathway could offer the most attractive commercial platform for indigo biosynthesis, and potentially have the highest chance for successful commercialization. It analyses which large-scale bioprocesses may be technically possible and which relevant process attributes could be achievable. The potential biological platform outputs are then calculated and assessed against the relevant facts from the indigo industry, to discuss their anticipated business viability.

## Overview of indigo biosynthesis and recorded production pathways

### Physicochemical properties of indigo

**Fig. 1:**
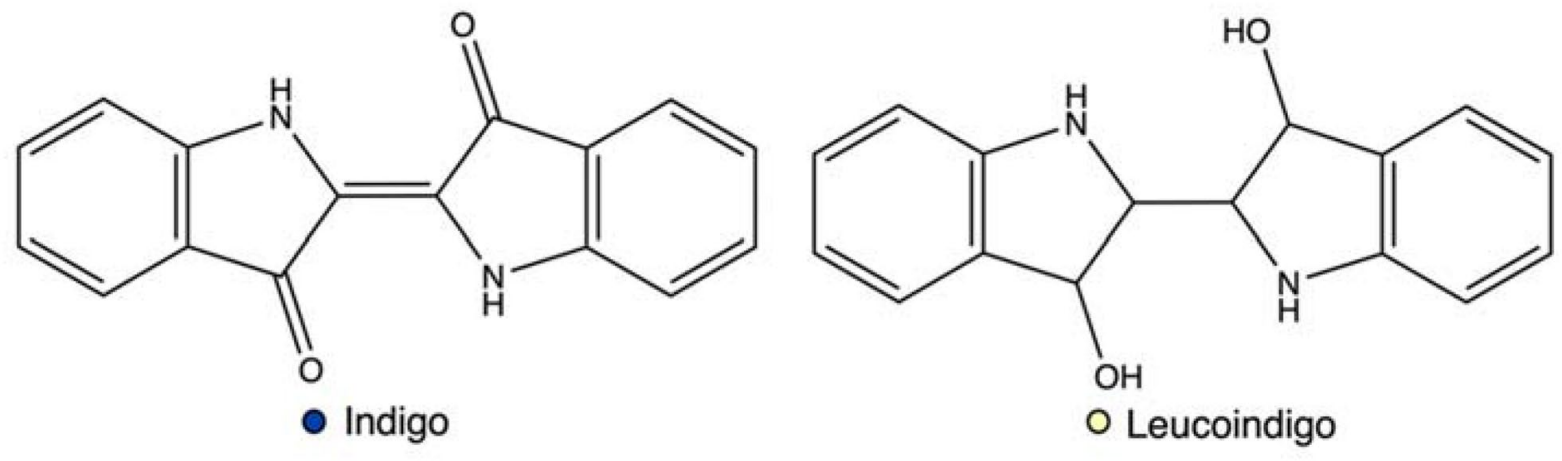
Structural formula of indigo and leucoindigo with corresponding compound colours

Indigo is an organic aromatic molecule with the chemical formula C_16_H_10_N_2_O_2_, which consists of two indole rings (Fig. 1). This influences the stoichiometric indexes and substrates’ ratios for indigo synthesis, as to produce 1 mol of the dye, 2 mol of its precursor are required. In both, the solid state and solution indigo exist in the trans form, and this isomer is maintained due to intramolecular hydrogen bonding between the adjacent amine and ketone groups (Głowacki et al. 2012). The stabilization from inter- and intramolecular hydrogen bonding is also responsible for other important properties: the high melting point (390–392 °C), which aids indigo’s stability as a dye, and the solubility properties. Indigo has only limited solubility in organic solvents and is insoluble in water, thus the compound is classified as a vat dye. For vat dyes, the dyeing originally was performed in a vat or a bucket and a reducing agent was added to solubilize the colouring substance (Balfour-Paul 2011). For indigo, only after the reduction and removal of oxygen in an alkali environment will the dye form a soluble, pale yellow leuco form, that may be used in dyeing activities. The deep blue tones reappear upon oxidation and the indigo is oxidised to the insoluble blue form and precipitated and trapped in the textile fibres. For effective adsorption of the leucoindigo onto cellulose fabric, the pH conditions must be suitable, as the compound may exist in solution in different leuco forms, from acidic to alkaline (Blackburn et al. 2009).

**Fig. 2:**
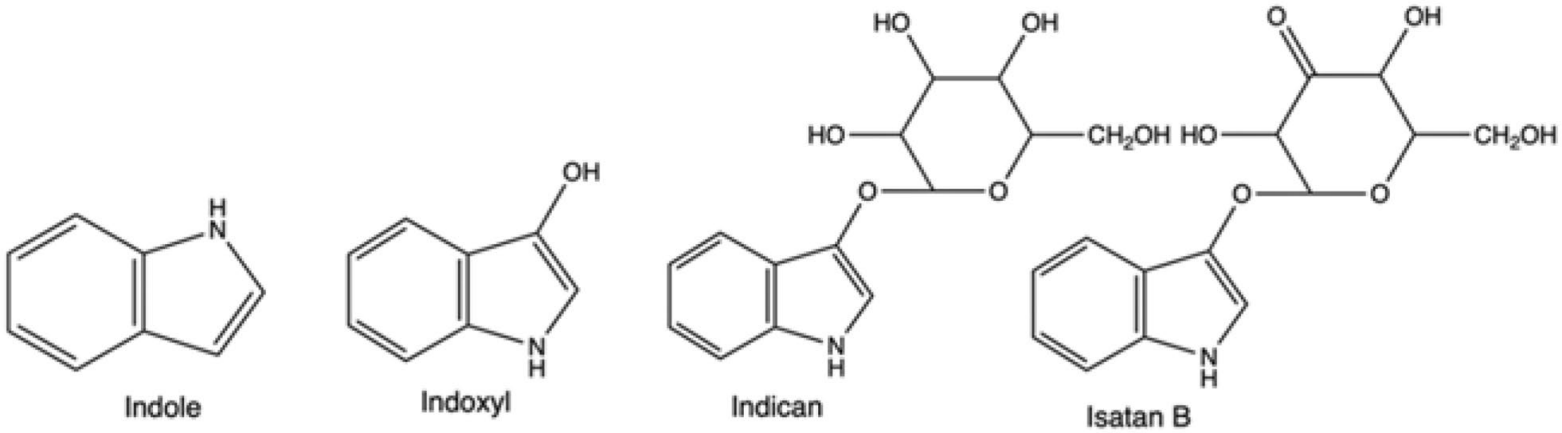
Structural formula of indole, indoxyl, indican and isatan B

Indigo is not present in nature itself; however, its natural precursors are found in several plant species. The direct indigo’s precursor, reactive indoxyl (Fig. 2), is usually attached to a glucose moiety and forms a glucoside, indican, in the plant leaves. Another plant precursor is isatan B, although nowadays, it has less economic importance for natural indigo production (See “Traditional plant-based production” section). On its own indoxyl is unstable, and thus after cleavage of sugar residue, it dimerizes and oxidizes in the air due to the presence of atmospheric oxygen and forms stable indigo. Indoxyl is a derivative of indole (Fig. 2)—a nitrogen heterocyclic aromatic compound, which is found in many places in the environment, especially plants and bacteria-rich materials, such as soil, sludge or intestinal tracts (de Sá Alves et al. 2009).

**Fig. 3:**
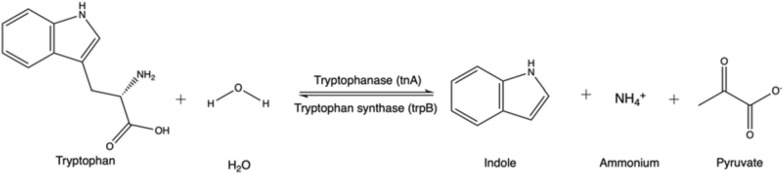
Reaction mechanism of indole production from tryptophan by enzyme tryptophanase

Lee and Lee (2010) published a review with 85 known different indole-producing bacteria, either Gram-positive or Gram-negative, that also have homologues of a relevant enzyme, the *E. coli* tryptophanase (TnaA) (Deeley and Yanofsky 1981). Indole may be produced by the action of these enzymes on the proteinogenic amino acid, tryptophan (Fig. 3).

### Indigo production pathways

This section provides an overview of the main production platforms for indigo. Besides academic groups, several commercial companies have shown interest in investigating the manufacture of this compound, including the biological approaches. Firms often keep internally developed processes as company and trade secrets so not all the steps used in the industry might be described here. Internal company knowledge gives them a competitive advantage and mitigates the rise of competition.

#### Traditional plant-based production

In nature, plant material, mainly leaves of different species, contains the organic precursors of indigo, often in glycosylated forms, such as the colourless indican. The corresponding indican metabolic pathway was solved relatively recently—indican is formed as a result of indole oxygenation at the C3 position to the reactive direct indigo precursor, indoxyl, and following this it is glycosylated (Inoue et al. 2017; Jin et al. 2022). Across the globe, several plant species contain indigo precursors, and human populations in different regions have discovered indigo extraction independently and at different time points (Balfour-Paul 2011; Paul et al. 2021).

In Europe, for centuries *Isatis tinctoria* (woad) has been cultivated for indigo production and was often a dominant industry in some regions and a source of its wealth. Głowacki et al. (2012). An example is the German Thuringia region, which was the centre of woad cultivation in Europe. Its great prosperity was reflected by the creation of one of the oldest European universities in Erfurt in the XIV century, which could maintain functioning also due to steady and significant income from the indigo trade (Berger and Sicker 2009). However, with the intensification of international trade, the import of Indian indigo eradicated woad-based extraction in the sixteenth century. The tropical and subtropical indigo-bearing species of *Indigofera*, especially *Indigofera tinctorium* (*I. tinctorium*) have a significantly higher content of single indigo precursor, indican, while woad contains lower amounts of two precursors: indican and isatan B (Gilbert et al. 2004). Thus, processing *I. Tinctorium* enabled higher yields and better cost-effectiveness.

**Fig. 4:**
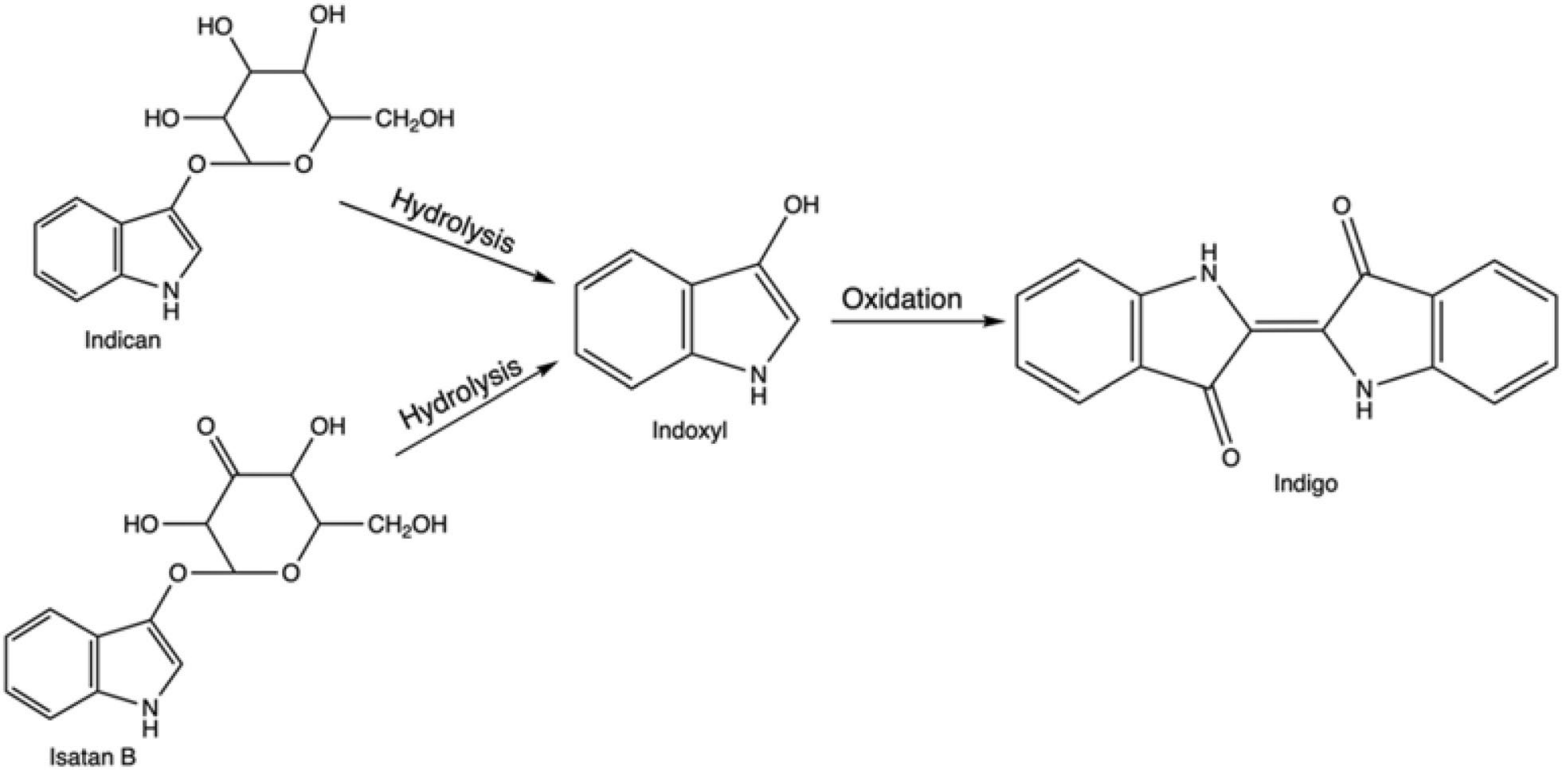
Natural pathway: from plant precursors, indican and isatan B, through intermediate indoxyl to indigo

Historically, indigo production commenced with fermenting material of a suitable plant in fermentation vats. The extraction of indigo precursors from biomass relied on the natural growth of aerobic and anaerobic bacteria, which were able to break down glucosides—the compounds derived from glucose (Blackburn et al. 2009). The bacterial consortium species would produce enzymes, that hydrolysed the indigo precursor into indoxyl and appropriate residue, for example, β-D-glucose, if indican was the substrate within the plant (Fabara and Fraaije 2020). In the next steps air would act as an oxidant, dimerizing it and resulting in obtaining solid indigo (Fig. 4).

The preference of dyeing units toward synthetic than plant-derived indigo may be explained by its much lower purity of 20–50%, or even 7–45%, when compared to 94–100% pure synthetic indigo (Shi et al. 2021; Wenner and Forkin 2017).

##### Should the plant-based platforms be revisited?

Although the traditional plant-based pathway was ultimately replaced by chemical synthesis in the nineteenth century, Fabara and Fraaije (2020) suggested that in line with the movement toward sustainable industrial processes, this pathway could be reconsidered. The modification of this route was enabled by relatively recent research on genetic insights into natural indigo synthesis pathways (Inoue et al. 2017; Jin et al. 2022).

Moreover, Fräbel et al. (2018) generated non-natural halogenated indigo precursors *in planta* with genetic engineering. This suggests that modifying and improving the effectiveness and yield of natural pathways to native indigo-bearing plants may be also possible, especially if recombinant biology tools would be applied. Several commercial companies are indeed revisiting the concept of plant-based platforms—it could be based on cultivating plants with beneficial traits, such as high-indican content or low soil quality requirements. Still, from business and commercial perspectives, much optimization would be needed for economically competitive manufacturing.

An example company is Stony Creek Colors, which has developed high-yield, high-crop leguminous indigo plants for Tennessee (US) farmers, as an alternative for tobacco plantations. The company recently partnered with Archroma, an international indigo supplier, to produce a plant-based pre-reduced indigo product at scale (Archroma 2022). Although this pathway offers a greener alternative to aniline-based indigo production, it is arguable if plant-based large-scale production is the most sustainable route. Producing 10 kg of indigo requires 1t biomass, and the indigo plantations would compete with food and feed production, an aspect which is almost absent for chemical platforms (Pattanaik et al. 2019). For example, Choi (2020) calculated that to produce 8000 tons of natural indigo, which is just 10% of the global annual demand (Additional file 1), 1.7 million acres of arable land is needed. Moreover, the textile industry is water-intensive and already consumes up to 9 trillion litres of water annually (Centre for Global Equity Equity 2021)—the plant-based indigo production platforms use 0.4% more fresh water than chemical platforms, as revealed by ISO 14040/44 standard Life Cycle Analysis (LCA) (FT Research Team 2018). In contrast, microbial fermentation would introduce water savings. Based on the information provided by PILI, a start-up commercialising microbial dyes (www.pili.bio), chemical production of indigo dye uses 1000 L water for 10 kg dye, giving dye to water ratio of 1:100, while PILI’s microbial platforms use 5 × less water, giving a better ratio of 1:20 (PILI 2021; Appendix). Another perspective, that makes microorganisms attractive, is given by start-up Colorifix (www.colorifix.com) which develops innovative microbial in situ dyeing platforms, where microbes directly fix the dye on the fabric (Centre for Global Equity Equity 2021). This solution might be difficult to fully implement within the textile industry, although it offers large water reduction during a different step—the dyeing procedure. Microbial in situ dyeing uses a 3:1 water to fabric ratio, while dyeing with plant or chemical dyes requires ratios of 10:1 for ‘best’ cases, although values up to 50:1 are more common (Ajioka and Yarkoni 2017).

#### Chemical synthesis of indigo

The chemical indigo synthesis was developed in the nineteenth century. The first pathways were not economically competitive to plant-based production; however, it changed after a significant (£1 million) investment from the German company BASF for research, as described by Głowacki et al. (2012). The BASF textile dyes unit had been the key indigo supplier until the 1990s, when it merged with similar units from other large companies, such as Bayer AG or Imperial Chemical Industries (ICI), forming a joint venture DyStar (DyStar 2021b).

**Fig. 5:**
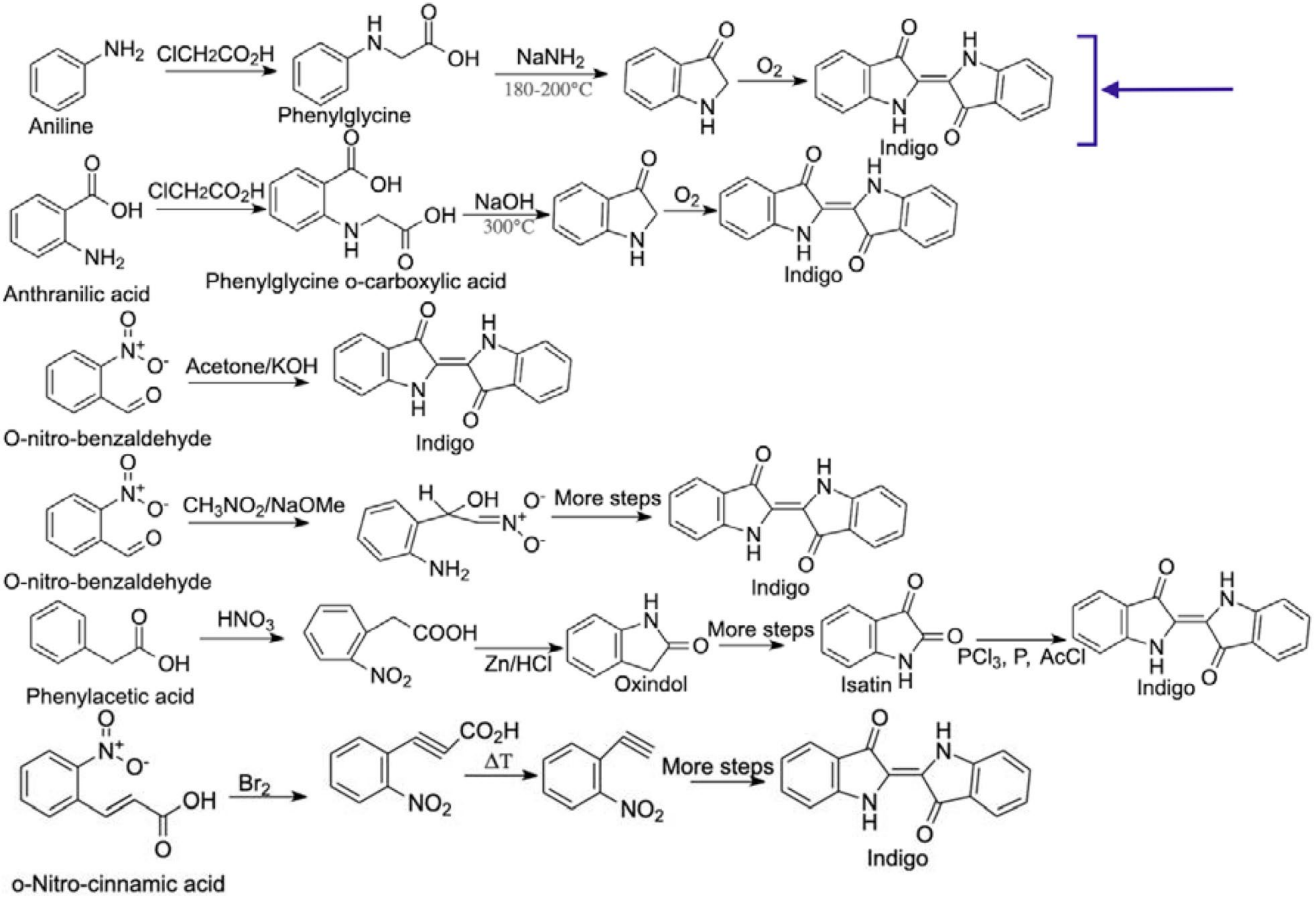
Representative chemical pathways toward synthetic indigo. The main route is highlighted with purple lines and an arrow

There are at least 6 different developed chemical pathways and each requires non-renewable aromatic substrates (Fig. 5). The first commercialized pathway was economically unsuccessful—it used expensive nitrocinnamic acid as key raw material (Baeyer and Drewsen 1882), while multiple process steps provided low indigo yields. The first commercially competitive and successful synthetic process used anthranilic acid (Schmidt 1997) and was later modified and improved to rely on aniline as a substrate (Pfleger 1901). It is the dominant process nowadays within the industry (Christie 2014) with the merits of high productivity, indigo purity, simplicity, and low cost (Paul et al. 2021; Schmidt 1997). Due to its prevalence within the dyeing industry (Paul et al. 2021), this pathway is assumed as the gold standard of synthetic indigo manufacturing, against which in this work the potential biological platforms will be compared, especially in “Overview of technically feasible bioprocesses and their contextual evaluation” section.

Aniline is an aromatic hydrocarbon that has to be firstly derived from benzene, for example, via the reduction of nitrobenzene by catalytic hydrogenation, which is a reaction that relies on hydrogen treatment in the presence of metal catalysts (Blaser et al. 2001). For the commercial synthesis of aniline, the reaction would often rely on nickel or copper (Gelder et al. 2005). With the use of hydrogen cyanide or cyanic acid and formaldehyde in a buffered bisulfite solution (phenylamino)acetonitrile is derived. This compound is then the starting material for the cyclisation reaction, which requires a mixture of NaOH/KOH and NaNH_2_ to give indoxyl (Blackburn et al. 2009; Chakraborty 2015). As at most stages of this chain of reaction the by-products are not separated, indoxyl firstly forms a metal salt, and is then (Mermod et al. 1986), dissolved in water to enable oxidation and formation of indigo and undergoes purification (See “Overview of technically feasible bioprocesses and their contextual evaluation” section; Paul et al. 2021).

As a leading textile dye, the industrial-scale production of synthetic indigo costed ~ 4 USD/kg (Głowacki et al. 2012). However, a major concern is the toxicity of substrates and waste products, and this cost is not accounted for in this figure yet.

##### Toxicity issues

The chemical synthesis of indigo is unsustainable and unfriendly to the environment. This creates a problem of large scale, as the global indigo demand and industrial-scale production require using large quantities of materials, creating large waste streams and disposal issues. Chemical processes use non-renewable petrochemical precursors and the majority of indigo comes from a few facilities in developing countries, where aniline is used as the key substrate. Aniline is very toxic to humans and is classified as a probable human carcinogen (Group B2) (U.S. HSDB 1993). Acute and chronic contact causes skin allergies and upper respiratory tract irritation, while repeated exposure may affect the oxygen transfer by red blood cells, leading to increased heart rate, headaches or unconsciousness (EPA 2000). Aniline is toxic to wild and aquatic life (EPA 1985) and its appearance on compound lists restricted for use in industry is increasing (ECHA 2021).

Internal studies of the above-mentioned Archroma, claimed minimally 400 tonnes of aniline escape annually from indigo production (Scott 2015). It suggested that 2/3 of this compound loss is divided between wastewater, air and plant workers, while 1/3 remains on the denim fabric distributed as commercial products. The finished textiles, dyed with synthetic indigo, were found to contain up to 2,000 ppm aniline, meaning that customers are directly exposed to this toxic compound. Archroma states that its technology of manufacturing pre-reduced indigo prevents the petrochemical substrate from carrying-through as a contaminant, although this is rather a partial solution.

Although aniline has been detected in both, the indigo dyes (Cordin et al. 2021) and informally in the finished jeans products (Warren 2019), there is no sufficient evidence to rule if these quantities are harmful to jeans wearers and to what extent. Still, the use of aniline as raw material imposes proven health hazards for the facility workers, as the aniline-poisoning and its complications are well-documented in the chemical industry, including the dye plants (Käfferlein et al. 2014; Kumar et al. 2014). Moreover, the presence of aniline in the process wastewater imposes the risk of run-off to larger water bodies, their subsequent contamination and danger for an increased number of people, as aniline impacts humans if ingested, inhaled, or dermally absorbed (Dutkiewicz and Piotrowski 1961; Korinth et al. 2012).

Usually, multiple hazardous, toxic chemicals are also used during indigo synthesis, mainly formaldehyde, hydrogen cyanide or cyanic acid, sodamide and reducing agents (in excess), and corrosive hydroxides, including NaOH or KOH (Paul et al. 2021). The latter is necessitated to enable dyeing fabrics, as indigo is insoluble in water and must be firstly reduced (“Physicochemical properties of indigo” section). These substances and their by-products may corrode equipment and pipes. Paul (2015) presented as an example sodium dithionite (Na_2_S_2_O_4_), commonly used due to its low price and short reduction time, which decomposes to sulfate and sulfite. A comprehensive list of other reducing agents and operations may be found in Paul et al. (2021), which includes, for example, several metal-containing compounds or formaldehyde sulfoxylates, although these further options are not as established nor common.

The established aniline chemical pathway toward indigo, which uses the mentioned chemicals and harsh and high-temperature conditions, relies on a chain of reactions and cyclization. The intermediate by-products are often not removed during the process, and studies involving analysis of commercially available indigo products reported the presence of substances, such as aniline, N-methylaniline or anthranilic acid (Cordin et al. 2021).

To avoid spending resources on wastewater treatments, many facilities dump the spent dyestuff directly into rivers, contributing to environmental pollution (Scott 2015). This could not happen in more developed and wealthier areas, such as the EU or US, where sustainability matters are addressed more thoroughly. Waste disposal is strictly regulated in the EU and US, especially in the context of toxic substances and hazardous chemicals. For example, all UK-based companies that produce these must register with the governmental Environment Agency for annual volumes over 500 kg (Hazardous Waste Regulations 2005). For a commercial business, this is a very low level, equivalent to, for example, 500 items of 4-L sharps bins (First Practice Management 2022).

However, out of recently reported 17 large industrial plants that produce indigo, only 1 was in Europe, while 16 were based in other countries—13 in Asia, and 2 in Middle and South America. This means 87% of indigo production is located in low-wage and lower-income countries (Paul et al. 2021). In those regions, the legislation does not prevent wastewater and the outflow of chemicals into the environment effectively.

These toxicity issues drive the need for a more sustainable platform, making indigo biosynthesis highly attractive.

#### Biological production of indigo

**Fig. 6:**
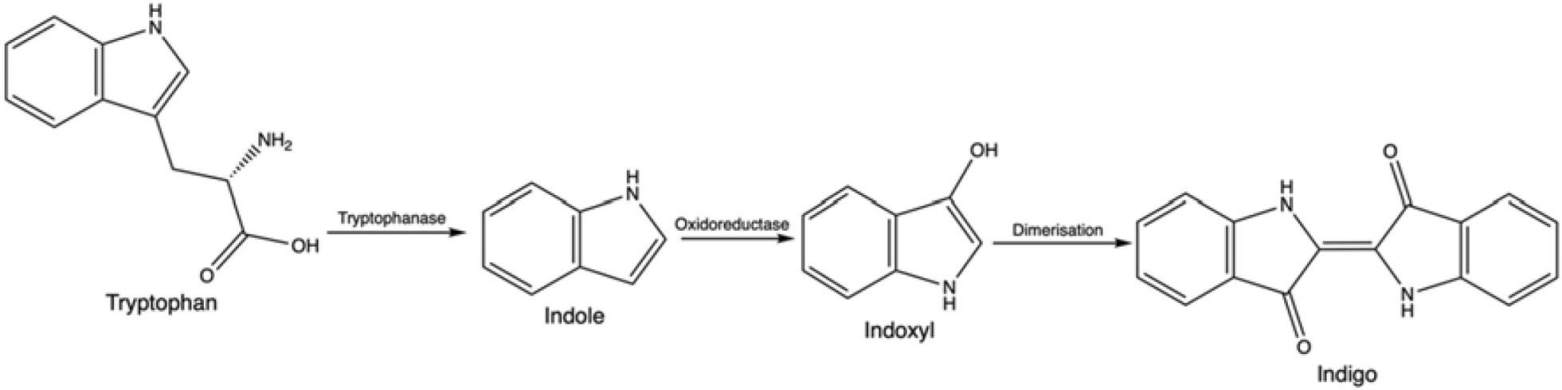
Generic metabolic pathway from tryptophan to indigo

The indigo precursor, indole, is widely distributed in the natural environment, and some microorganisms have the ability to transform or degrade this compound (Ma et al. 2018). The investigation of indigo biosynthesis with bacteria was initiated by the fortuitous discovery of Ensley et al. (1983) from Genencor, where during heterologous expression of the naphthalene dioxygenase (NDO) enzyme system from *Pseudomonas putida* G7 containing the plasmid NAH7, colonies of the host organism, *Escherichia coli*, turned blue. The initial substrate is tryptophan from the medium, on which the *E. coli* enzyme tryptophanase acts, leading to the formation of endogenous indole (Fig. 6). The key pathway step is indole oxidation by the NDO enzyme system via the addition of oxygen to indole and formation of the direct indigo precursor, indoxyl, which then spontaneously dimerizes to indigo. The relevant enzyme class for this final reaction are oxidoreductases (EC 1.).

Most wild-type microorganisms that could potentially produce indigo, as they possess oxidoreductases with broad enough specificity to hydroxylate indole, are hydrocarbon-degrading bacteria species (Bhushan et al. 2000), especially from the *Pseudomonas* genus (Ma et al. 2018). However, research work often pioneered *E. coli* as the organism to host the recombinant oxygenases (Berry et al 2002; Murdock et al. 1993), largely due to its establishment for the production of various metabolic products (Patnaik and Liao 1994) and the availability of native tryptophanase, TnaA.

Since the initial discoveries, many indigo-producing oxidoreductases have been identified, often accidentally during observations of particular microbial strains, when researchers noticed unusual pink and blue tones of colonies when grown with indole. More recently, genome and metagenome mining were used to investigate further possible enzyme candidates (Lim et al. 2005; van Hellemond et al. 2007; Nagayama et al. 2015; Singh et al. 2010). Indigo formation is noticeable visually, thus its biosynthesis can be screened simply from clone libraries. Many studies specifically looked for oxygenases for indole bioconversion, including mono- and dioxygenases, as these offer importantly chemo-, regio-, and/or enantioselectivity, that often cannot be rivalled by chemical approaches (Urlacher and Schmid 2006).

Crocker and Fruk (2019) also reported the possibility of light-driven oxidation of indole to indigo. The methodology relied on a monooxygenase enzyme and a flavin-polydopamine (FLPDA), that was activated under blue irradiation and ambient conditions. light-driven monooxygenase. This bypassed the use of external reducing agents, such as NADH, although with the complexity of FLPDA synthesis the application of this method for the industrial production of indigo could be challenging. It is also worth noting that not all experiments, that harnessed indigo bioconversion, targeted indigo production per se. Formation of indigo has been also used as a tool itself, for example, as a colourimetric screen to find oxygenases. The reason is that positive and negative outcomes can be easily distinguished with the naked eye by appearance or lack of blue hue.

A large concern is the much lower productivity of identified microbial pathways when compared to chemical synthesis (Pathak and Madamwar 2010), thus new enzymes and indigo-producing microorganisms are looked for, and known pathways are optimised and modified. For example, Ensley et al. (1983) found indigo bioconversion is boosted by adding tryptophan or indole to medium, although 10 more years had to pass before employees of another company, Amgen, combined a few metabolic engineering modifications and constructed an indigo pathway from glucose in *E. coli* (Murdock et al. 1993).

Most available publications considered the biological indigo pathways at a small-scale, while the transformation of a biological reaction into a cost-effective large-scale bioprocess often requires analysis of several, additional different aspects because of the nature of the biological entities. Significant consideration and resources have to be devoted to minimising the output variability of a bioprocess system, which, for example, has no place in purely chemical or mechanical platforms. However, with the rise of control and monitoring of the process, the operation costs rise, while also as the process scale increases, maintaining constant parameters becomes more challenging. At a bench scale the components are usually mixed without large hurdles, while processing several hundred litres takes longer, requires special equipment and is difficult due to the viscosity of cell cultures and often complex solids separation. Laboratory-scale experiments may also use very pure or expensive components, which after the increase in quantity during scale-up could easily become infeasible or impractical (Cacciuttolo and Arunakumari 2005). Thus, before cost-effectiveness evaluation, the practicability and technology possibilities of industrial-scale indigo biosynthesis execution must be considered.

## Methodology

We consider here the approaches that were used to investigate the biological production of indigo and the aspects assessed as relevant to this subject. Predicted limitations of the pursued methods are also included.

### Investigation of indigo biosynthesis and relevant aspects

The approach relied mainly on literature search and analysis. The suitable sources originated largely from academic and research environments, and were accessed with search engines, mainly PubMed (https://pubmed.ncbi.nlm.nih.gov/) and Google Scholar (https://scholar.google.com/); and online libraries, such as Wiley (https://onlinelibrary.wiley.com/) and Science Direct by Elsevier (https://www.sciencedirect.com/).

In parallel, companies, research groups and individual scientists, whose work has been associated with topics relevant to the review subject, were identified, mainly in biocatalysis and recombinant production of valuable small molecule compounds by bacteria. Then, the information, studies and sources connected with these parties were explored to avoid omitting disclosed, accessible relevant information due to aspects, such as word rephrasing or vague titles. For industrial parties and vendors, the sources included company websites, press releases, technical reviews or statements made by executives or authorities involved in relevant subjects. These included, for example, provision of the relevant regulatory framework and economical information, such as dye prices or legislation for chemical waste disposal.

### Bioprocess design and analysis

With the information obtained in 2.1. potential large-scale indigo biosynthesis was considered, including upstream and downstream operations. The suitable flowsheets for indigo production and purification were constructed and evaluated to analyze, which unit operation could provide a commercial product of sufficient quality and characteristics, such as purity or formulation.

It was attempted to analyze if a biological platform could offer a competitive business opportunity, that could be attractive for the current dyeing industry by large-scale manufacturers. The different potential platforms for indigo production were proposed, evaluated, and compared based on relevant factors from cases of real-life facilities that use indigo for denim dyeing, notably Chinese- and Pakistani denim mills (See “Platform output analysis” section). Data such as annual indigo consumption and dyed yearn output enabled benchmarking in “Overview of technically feasible bioprocesses and their contextual evaluation” section , such as the production scale, that a novel biological platform would have to achieve. For simplicity, all considerations assumed monoproduct plants.

To project the annual output of a feasible biological platform, necessary assumptions were stated basing on the available circumstantial factors and information. Most values were fixed after estimation to enable output comparisons, although considerations were also performed for cases, where a single value of the proposed biological platform would change.

When there was a lack of precisely stated data, the necessary figures were deduced based on suitable justification and equivalent small molecule production values, such as common purification yields for bacterial bioprocesses.

The potential biological platforms are placed in the context of the most prevalent synthetic indigo manufacturing pathway, which has been designed by Pfleger and relies on aniline as the key raw material (See “Chemical synthesis of indigo” section) and generally these 2 approaches are compared against each other where relevant, especially in “Overview of technically feasible bioprocesses and their contextual evaluation” section .

### Limitations

The listed methodology enabled an in-depth investigation of recombinant indigo biosynthesis, although few factors impose certain limitations. An important issue is a restricted access to relevant data, objectiveness and justifiability of some, especially from commercial sources. The incentives of business competition and striving for profitability suggest, that companies may give selective or not fully objective information. objective information. Thus, the modelling results presented in this work should be considered bearing in mind the stated assumptions and limited publicly available information.

The industrial environment often also does not disclose technical expertise, which for indigo and denim dyeing mostly resides exclusively in production sites and companies (Paul 2015). Across the project, some assumptions had to be made to enable results comparison. Within a business environment, even the published information is often worded vaguely and inexact, for example, patent content. It impedes finding the data and its use by competition for informational purposes, such as understanding the research areas of the competitor (Dehns 2018). Due to this practice, relevant information could be omitted, although actions were implemented to mitigate this (“Upstream processing (USP) considerations” section).

## Overview of technically feasible bioprocesses and their contextual evaluation

### Designing an industrial-scale biocatalysis

Within the bioprocess engineering sector it is said that “the product is the process” (Doran 1995)—to assess commercialisation’s viability, both, the holistic big picture of the platform, and its details, must be considered. Such evaluations for many biologically produced compounds are published, including high-value commodities, such as antibodies (Farid 2007) and lower-value, for example, different organic acids (González et al. 2007). However, no considerations for indigo biosynthesis are available, and this section aims to address that. This section comments on important design aspects of a possible large-scale platform, including relevant upstream parameters and the required plant capacities for an industrially attractive biological platform, that was based on the current real-life indigo supply demands of the commercial dyeing units.

It then proposes 2 main different bioprocess sequences (flowsheets), based on the physicochemical properties of indigo and specifications of the potential commercial product. Then, the major chosen bioprocess unit operations are discussed, and their rationale is provided. In parallel, the current chemical manufacturing process is also presented, as well as a potential *in-situ* biological platform, that reflects recent trends toward a combination of the bacterial production of a relevant enzyme and dyeing procedure in a single step.

**Fig. 7:**
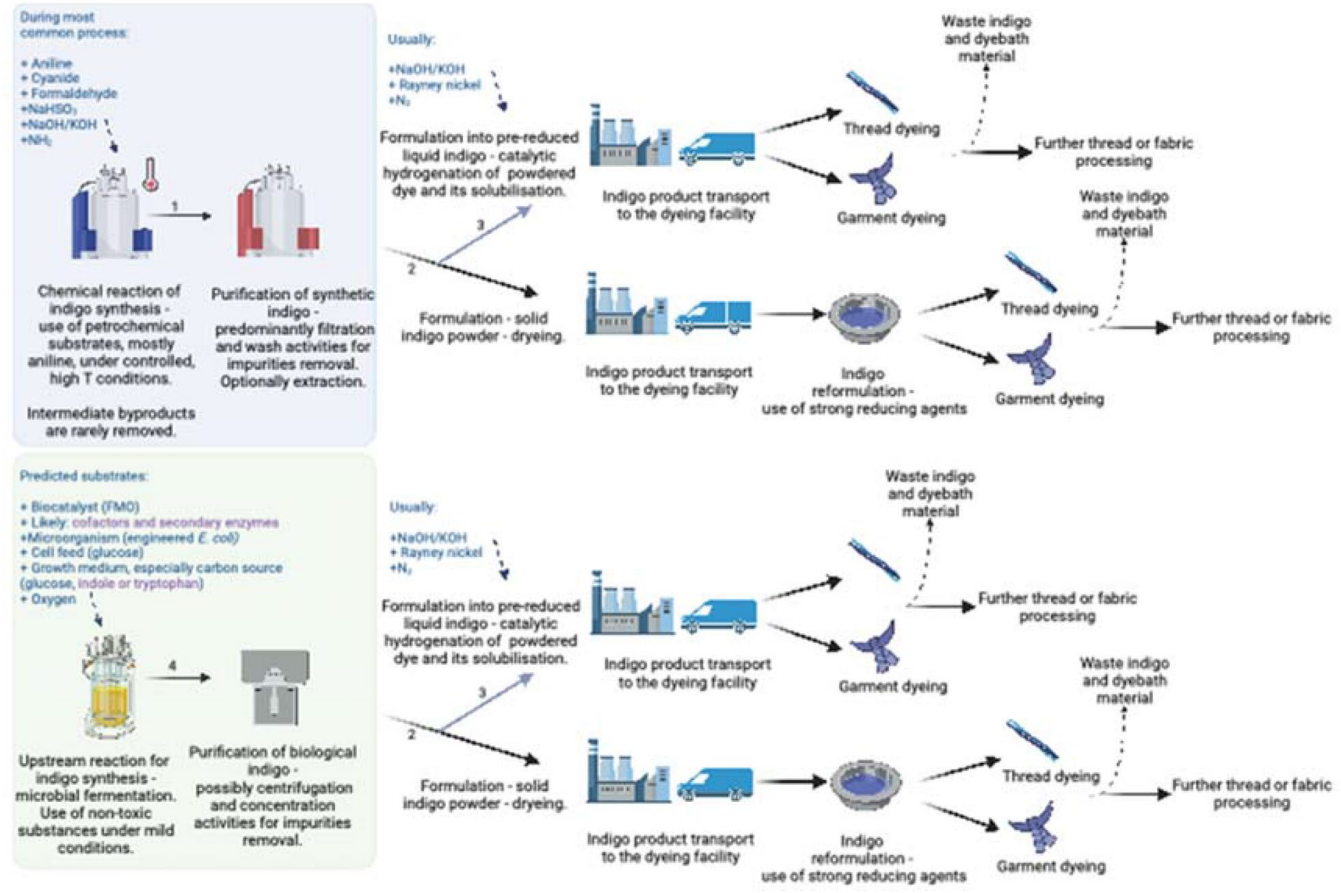
General flow diagram of the supply chain of the chemical manufacturing of 2 main indigo products, a powdered dye, or a liquid pre-reduced solution. The light blue area marks the steps that would be different if a biological manufacturing platform would be employed, and these alternative operations may be found in the light green area. The final processing steps of the formulation are anticipated as similar to the chemical and biological platforms; therefore, they have not been included in the blue or green shading. ‘High T’ and a thermometer denote high temperature. The splitting arrows, numbered 2. (light blue) and 3., have been used to visualize the formulation of liquid indigo, which usually commences after the drying process has been performed to a certain extent. The numbered steps denote: 1.—the main chemical reaction chain toward the synthesis of synthetic indigo; nowadays in the majority of industrial factories, the core reaction relies on aniline or its precursors as the raw materials. 2.—the formulation toward powdered indigo; usually performed via drying. 3.—the formulation toward pre-reduced liquid indigo; usually performed via catalytic hydrogenation and the addition of strong reducing agents. 4.—the fermentation reaction that leads to the synthesis of biological indigo. The grey centrifuge represents the downstream processing sequence for product purification, for which the individual unit operations have been described in detail in “Downstream processing considerations” section. The dark blue writing represents the main substrates that are required to perform a certain manufacturing step, while the violet writing has been used to underline the potentially most expensive compounds. The figure has been created with the help of BioRender

To visualize and present the main steps involved in the process of indigo synthesis, Fig. 7 has been created. Its purpose is to provide a comprehensive overview of the main necessary actions that must take place, the main substrates and inputs needed. Each of both, the traditional chemical platform and the anticipated biological one, has been broken down into individual operations and placed next to each other, so that the precise differences between them may be conveniently compared. For the detailed description and reasoning behind the Figure elements, please see the Fig. 7 description.

Figure 7 spans the steps from the initial chemical or biological reaction of product synthesis to the ultimate use of mentioned commodities in industrial denim dyeing activities. The main source for the information relevant to the chemical processes has been Paul et al. (2021), while the biological platform has been created based on the following work and considerations presented in this document.

The currently employed chemical platforms commence with a chain of chemical reactions, with the majority of facilities relying on aniline, or its precursors, as the main raw material. Next, the formulation of indigo follows, which usually relies primarily on drying for the standard dry powder products. For the liquid pre-reduced commodities, drying is usually performed to a certain threshold, and then catalytic hydrogenation or strong reducing agents are employed to derive a leuco indigo solution. This operation usually relies on first drying the indigo and forming the dye into a powder, and this powder is then taken, exposed to catalytic hydrogenation, and dissolved. For a more detailed description of the bioprocess sequence and purification steps see Fig. 10 and Section "Proposed process flowsheets".

The major differentiation of the designed biological platform for indigo production would occur at the initial stages, as the chain of chemical reactions would be replaced by microbial fermentation and associated relevant biological molecular processes. It was decided to highlight especially in this novel biological platform the most expensive reagents and compounds used (marked with violet font) solution, as these could impact the cost-effectiveness of the platform, and hinder its implementation. In comparison, the chemical commodities, on which the traditional pathway (blue area) relies, are generally cheap. Moreover, for the chemical platforms, the most common process parameters and pathways have been widely established and are known, and the commercial viability is proven by multiple real-life examples and the existence of the whole synthetic indigo industry. For biological platforms, the optimal conditions and substrates are currently propositions and would have to be validated, and thus have been placed in brackets, while the word ‘likely’ relates to the fact that the requirement for secondary enzymes and cofactors will depend on the chosen the main biocatalyst, that catalyzes the oxidation of indole into indoxyl, such as the FMO.

### Upstream processing (USP) considerations

#### Use of whole-cell system vs. purified enzyme

The bioprocesses that rely on enzymes, such as indole bioconversion to indigo, may use a purified biocatalyst solution, or a whole-cell system, and this design carries important consequences. When a host organism is used, the optimization of the reaction of interest is more difficult, due to the complexity of cellular networks and their microenvironment (Cossar 2019). However, the direct process-related expenses are usually lower for the whole cell system and production, while the organism is growing. To use a purified enzyme, this biocatalyst must be separately produced and purified *in-house*, or a suitably pure solution ordered. Both options often generate substantial costs.

**Fig. 8:**
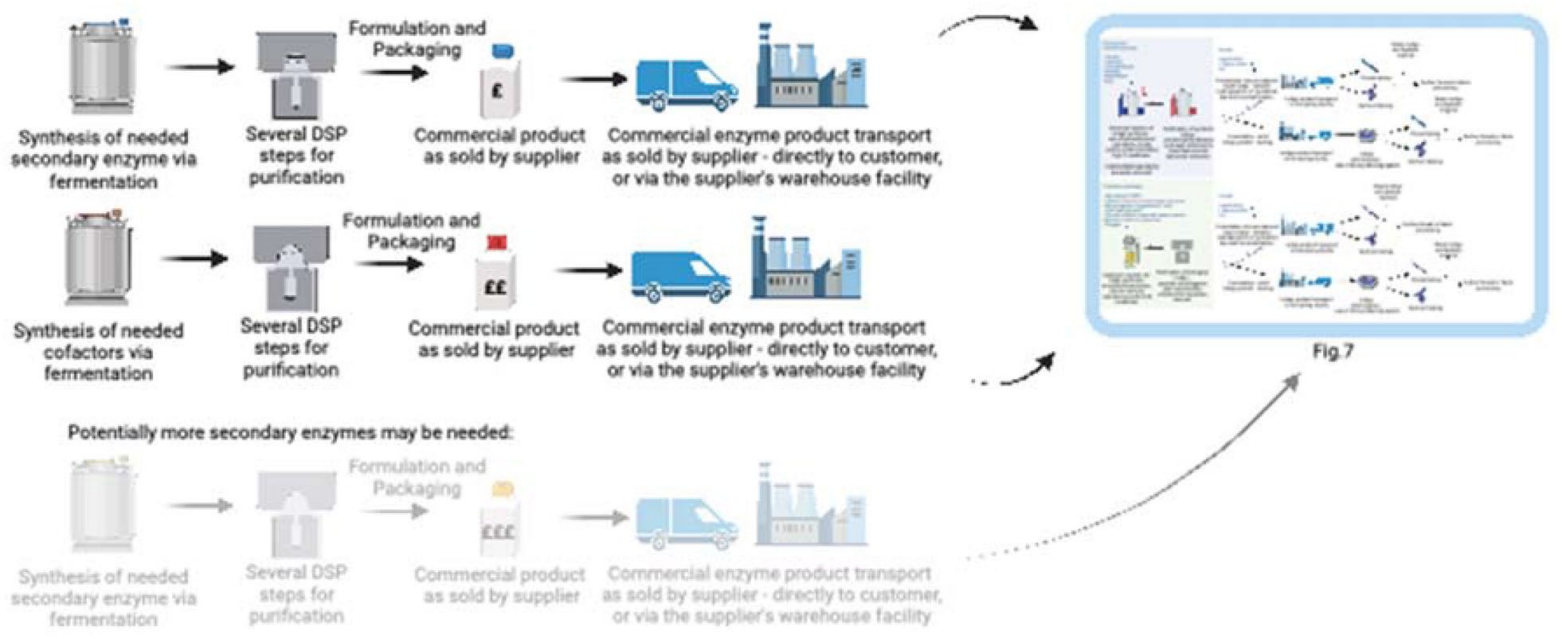
Representation of the supply chain extension of processing of the anticipated most expensive compounds required for the synthesis of biological indigo

For some reactions it may be very challenging to design an effective process without a cell host—many enzymes rely on cofactors and electron donors to perform the reaction (Renata et al. 2015). For most oxidoreductases, relevant for indigo biosynthesis, the oxygenation is catalysed by the provision of additional electron-donating cofactors, such as FAD or NADH, molecular oxygen, O_2_ and usually other proteins and enzymes are involved. Likely, the platform would have to include a system enabling the recycling of the electrons, for example, another auxiliary enzyme (Monti et al. 2011). Thus, at least two or three biocatalysts would need to be separately obtained, purified, and transported to the facility (if ordered), very likely steeply increasing process costs, before these substances can be used for the synthesis of biological indigo (Fig. 8).

#### Substrate costs—a viability driver of indigo biosynthesis

At an industrial scale, the cost of goods (COG)—the starting, raw materials—is one of the main drivers of a processes’ feasibility, next to the yield of the reaction of interest (Wu et al. 2021). The past attempts to commercialize the biological synthesis of indigo were hindered by the high cost of starting materials—tryptophan and indole (Berry et al 2002). Technically, a process, which would mimic largely the current chemical processes, where an isolated oxidoreductase solution is used on raw indole, could be executed. However, any platform that would use either indole or tryptophan, as the main raw material, would be difficult to design in a feasible and profitable manner due to their high prices. To roughly visualize and confirm this trend, the prices offered by 3 established media suppliers: Sigma-Aldrich/Merck, ThermoFisher Scientific and Avantor by VWR, for several commercially available products tryptophan, indole, and glucose, have been analyzed. Different possible quantities have been included in the calculations to account for the fact that the larger the ordered product amount, the better price may be offered by the supplier for a particular commodity. Still, this trend is not infinite, and the prices will fall to a certain extent and the differences between lower and higher value commodities can be spotted. Here, the average 1 kg price has been calculated for tryptophan, indole, and glucose—when compared to glucose, tryptophan is more than 11 × the cost of glucose, while for indole it is nearly 4x (Avantor 2022; Sigma-Aldrich 2022; ThermoFisher Scientific 2022; See Additional file 1: Excel Spreadsheet 1 for Calculations). For more process economics estimations, see “Overview of technically feasible bioprocesses and their contextual evaluation” section.

In 1993, Murdock with co-workers engineered a recombinant de novo metabolic pathway to synthesize indole and indigo from glucose, which opened the path to more viable fermentation, as glucose is a relatively much cheaper substrate. The key step of indigo biosynthesis is the indole oxidation to indoxyl, and a large-scale bioprocess would need to provide an effective platform for this reaction.

Thus, two main platform designs are proposed as worth investigating:

1. A whole cell catalysis, where cells produce from a cheap feed, such as glucose, the tryptophan and then indole, and a purified oxidoreductase is added.
2. A whole cell catalysis, where cells produce from cheap feed, such as glucose, the tryptophan and then indole, and the host expresses an oxidoreductase—the whole metabolic pathway happens in a single organism.

**Fig. 9:**
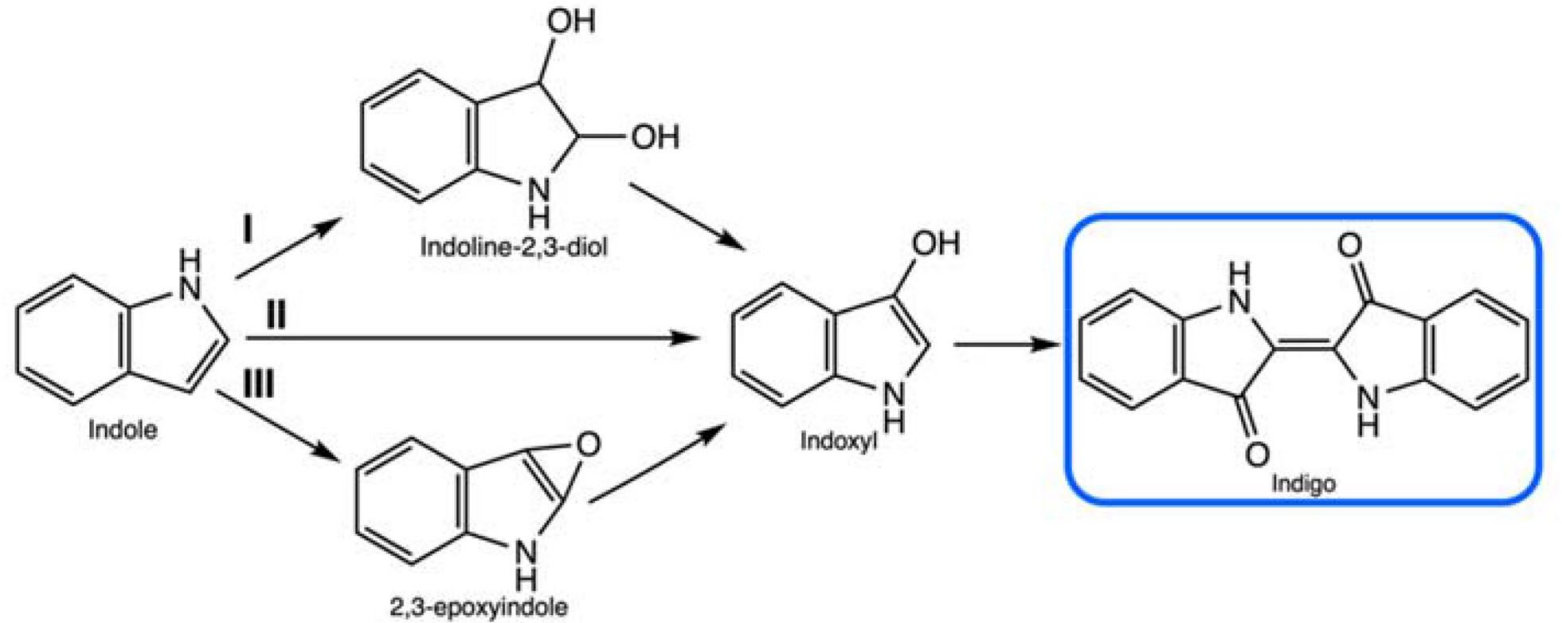
Different enzymatic indole pathways toward indigo: dioxygenation (I), direct hydroxylation (II), and epoxidation (III)

Currently, 3 different indole oxidation pathways are known—dioxygenation, direct hydroxylation and epoxidation (Fig. 9). A comprehensive review by Fabara and Fraaije (2020) presented currently known indigo-producing enzymes. Based on the cofactor used by the redox enzyme to oxygenate indole, these biocatalysts have been divided into 3 main classes: non-heme iron oxygenases, heme-containing oxygenases and flavin-dependent monooxygenases,

The fermentation reaction and process design that would be viable, depends on the available biocatalysts and their features. Thus, the oxidoreductases, which have been reported by literature for indigo biosynthesis, will be concisely summarised, alongside their important relevant characteristics. More detailed information is available in the work by Ma et al. (2018) and Fabara and Fraaije (2020).

It was mentioned in “Biological production of indigo” section, that production of indigo in bacterial colonies of the cloned genome or degradative plasmid fragments may serve as a colour screen for the presence of a novel dioxygenase. However, most groups were interested in the cloning of new oxygenases and not in the production of indigo for its own sake as a dye molecule. Later other groups showed that mono-oxygenases and hydroxylases would also act on the endogenous indole in *E. coli* and produce indigo. This expanded the scope of using the host and its internal indole as the screening tool for both novel oxygenase cloning and also for screening mutant libraries of recombinant oxygenases. As a result of the mentioned body of research, there are 5 main classes of oxygenase enzymes that can be used for the conversion of internal indole in *E. coli* to indigo and are potential candidates to consider for large-scale production of biological indigo.

##### NDO

A well-investigated enzyme for indigo bioconversion is the naphthalene dioxygenase (NDO; EC 1.14.13.8), which was the first discovered biocatalyst capable to catalyze this reaction (Ensley et al. 1983). It enables reaction via a labile intermediate, indoline-2,3, -diol, which quickly decomposes to form indoxyl and subsequently, indigo is formed upon dimerization (Pathway A). Several NDO homologs are known, that vary in substrate scope and were shown to produce indigo. Examples of such dioxygenases are toluene dioxygenase (Woo et al. 2000) and cumene dioxygenase (Groeneveld et al. 2016), although the bioconversion rates for indole to indigo were not investigated in much detail.

In addition to a heterohexameric oxygenase element, the NDO is composed of a flavin-dependent reductase and a ferrodoxin component, needed to transfer electrons from NADH to the oxygenase (Parales et al. 2000). Thus, for a functional NDO expression, 4 genes must be co-expressed—thus as a complex, multicomponent system, it might be not an ideal biocatalyst. Moreover, although with expression tuning and medium optimization the reported NDO yields reached 300 mg/L indigo in recombinant *E*. *coli,* other enzymes, such as monooxygenases, may offer twice as much yield (Fabara and Fraaije 2020).

##### mPHs

Bacterial multicomponent phenol hydroxylases (mPHs; EC 1.14.13.7.) are able to hydroxylate indole to indoxyl (Fig. 9, Pathway B) and may act on indole derivatives. McClay et al. (2005) via mutagenesis obtained enzyme mutants with different hydroxylation regioselectivities, each of which enabled the formation of different indigoid pigments.

Not many studies have been conducted on indigo bioconversion by mPHs, likely due to their relatively recent discovery. In the native bacterium, *Acinetobacter* sp. ST-550, a good yield was achieved (292 mg/L) (Doukyu et al. 2002), although in recombinant hosts the productivity was much lower—52 mg/ml (Doukyu et al. 2003). Still, similar to the NDO, the optimization of reaction conditions for using the cell-free mPHs may be challenging due to its complexity—these enzymes consist of 3 subunits: oxygenase element, flavoprotein reductase, required to generate and deliver electrons, and a cofactorless regulatory protein (Nordlund et al. 1990). Moreover, before designing a larger scale process, the optimization for the recombinant host would have to be conducted. The native bacteria, *Acinetobacter*, is used within the industry, although rather sporadically, and additional considerations would be required—for example, many of these strains do not use glucose (Lee et al. 2017).

##### P450s

Cytochrome P450 monooxygenases (CYPs; EC 1.14.14.X) were also studied for indigo biosynthesis. These enzymes possess heme as a redox cofactor and catalyze various oxidation reactions. Few human CYPs lead to indoxyl formation via Pathway B (Fig. 9), although due to eukaryotic origin and slow bioconversion rates these are not suitable for industrial processes (Gillam et al., 2000; Banoglu et al. 2001). There is a large number of bacterial CYPs, which are soluble proteins and these usually require additionally 2 soluble partners—a ferrodoxin and a ferrodoxin reductase (Hannemann et al. 2007). However, the case is different for so-called self-sufficient (SSF) CYPs, such as mutated microbial *Bacillus megaterium* enzyme P450BM3 (CYP102A1) (Li et al. 2000) or few natural biocatalysts (Fiorentini et al. 2018; Kim et al. 2018)—this was deemed very attractive and worth considering by Fabara and Fraaije (2020). SSP CYPs have a flavin-containing reductase besides the monooxygenase element and require NADPH, but not a secondary reductase, like NDO and mPHs, suggesting potentially simpler optimization and design of an industrial process.

##### UPOs

Novozymes employees patented the use of fungal unspecific peroxygenases (UPOs; EC 1.11.2.1) for indole and indole derivatives conversion—the researchers succeeded also in synthesizing the expensive indigo derivative Tyrian purple (6,6-dibromoindigo) (Kalum et al. 2014). For oxygenation, the extracellular UPOs source the oxygen and electrons indirectly, from hydrogen peroxide (H_2_O_2_), while all previously described enzymes depend on the provision of reduced coenzymes and cofactors, such as NADH, NADPH or FAD, that are generally expensive. However, after the reaction (Fig. 9, Pathway C), besides indigo, a side product 2-oxindole is formed, and UPOs may become inactivated if the H_2_O_2_ level is unsuitable. UPOs appear interesting for future indigo biosynthesis, after the aforementioned challenges are addressed, for example, via enzyme engineering. In addition, a few mammalian heme-containing enzymes acted as UPOs when given indole and H_2_O_2_, although side products were still formed and bioconversion rates remained low (Kuo and Mauk 2012; Barrios et al. 2014; Fabara and Fraaije 2020). Ullrich et al. (2021) developed a one-step synthesis of Tyrian Purple and several indigoids with 5 different wild-type and recombinant UPOs, that were selected from an earlier screening of 83 peroxygenases. The experiment involved also a 10L *in-situ* dyeing activity, although the need for process optimization before its commercialization has been underlined by the authors.

It must be noted, that due to an active Novozymes patent (Kalum et al. 2014), commercial exploitation of UPOs would have to include expenses for licensing out the technology.

##### FMOs

Many representatives from flavin-dependent monooxygenases convert indole to indoxyl, from 4 separate sub-classes. Many are two-component systems, containing the oxygenase element and a separate reductase, although several enzymes have fused genes for these. Such modification simplifies the biocatalysis process, and mostly these would be considered for the development of commercial bioprocesses. Especially the bacterial flavin-containing representatives (FMOs) are attractive, due to the highest reported reaction yields (Ma et al. 2018) (excluding the study by Berry et al. (2002), when the tryptophan pathway was severely modified for an NDO system). FMOs rely on NADPH and O_2_ and Han et al. (2011) achieved this with marine *Methylophaga* sp. FMO indigo from 2 g fed tryptophan in recombinant *E. coli* 911 mg/ml on a pilot scale (3000 L batch), while Ameria et al. (2015) reported a yield of 685 mg/ml for *Corynebacterium* sp. FMO. A start-up company, Huue is also already using an FMO for indigo biosynthesis via indican, as suggested by a paper published by one of its co-founders, Tammy Hsu (Hsu et al. 2018). Moreover, mutants of Baeyer–Villiger monooxygenases (BVMO) which may perform a similar reaction mechanism as FMOs, were also found to produce indoxyl and indigo (Pazmiño et al. 2007; Catucci et al. 2022). The enzyme appears interesting for bioprocess applications. It is thermostable—Fraaije et al. (2005) performed the biocatalysis at 52 °C—and tolerant toward organic solvents (De Gonzalo et al. 2006). Other flavin-dependent monooxygenases, that have been reported to produce indigo, including aromatic monooxygenases, such as indole or xylene monooxygenases (Dai et al. 2019), have been investigated by fewer studies.

### Downstream processing (DSP) considerations

After the upstream processing and biological indigo synthesis, the compound would have to be purified from the media and broth solution, to a level acceptable for its commercial sale and intended use. Many aspects may influence the DSP processing design, and these will be considered in this section.

#### Compound location after production: the cell, periplasm or media

The bioprocess design is influenced by the compound location after the reaction. After production by the cells the product may remain within them (intracellular production), be secreted into the medium (extracellular production) or, for some bacterial species, be accumulated within the periplasm (Tan et al. 2002). From the major two former cases, extracellular production is usually more desirable from the product recovery context. Otherwise, cells have to be disrupted, often by high-pressure mechanical homogenization (Nesbeth et al. 2012). This operation releases all cell content into feedstock, including high molecular weight DNA and various molecules, and results in high-viscosity, and -contamination homogenate. This necessitates more extensive purification increasing its duration for each batch and also the associated costs (Agerkvist and Enfors 1990; Nesbeth et al. 2012; Harrison et al. 2015). For some reported cases strains may be engineered to increase the product secretion into medium, although this is often recognized as a difficult and delicate to optimize task (Tan et al. 2002).

In the third case, if the product is periplasmic, there is an advantage of potentially not requiring a full cells’ disruption. Mechanical homogenization can be replaced by a milder operation, such as enzymatic or chemical lysis. Combining such operation with suitable upstream engineering, for example, co-expression of a nuclease to hydrolyze unwanted host DNA (Balasundaram et al. 2009), may reduce the viscosity of the homogenate feedstock, and enable more effective clarification and purification (Kelly and Muske 2004; Nesbeth et al. 2012).

The translocation of the compound of interest to the periplasm is heavily reliant on the nature of the product of interest. For proteins, this can be achieved by suitable plasmid and promotor engineering and influencing the trafficking activities of the cell (Robinson et al. 2011). However, for small molecules, such as indigo, there is much case-by-case variation. There is no universal transporting system that can be used for all such commodities, and each may need a unique and specific small molecule transporter system in the cytoplasmic membrane. This would be a transporter protein, that would help move the compound from the cytoplasm to the periplasm. Still, the additional genetic manipulation may lead to an increased metabolic burden of the exogenous plasmid on the cell and decrease the reaction efficiency (Schofield et al. 2016). Moreover, a relevant, effective, and specific transporter has not yet been identified for indigo translocation into the periplasmic space.

Although the compound location after reaction may be altered via bioengineering, primarily its location is always heavily influenced by the choice of microbial host for the reaction, what rules what will be more easily achievable or practically possible. For example, for *E. coli* trafficking of the compound into the periplasm might be achieved, while for mammalian cells not, as these cells do not possess periplasmic space.

#### Purification requirements—comparison with products of similar characteristics

Many different commodities may be produced via fermentation, of largely different structural and chemical characteristics (Kawasaki 2015). These are often categorized as either high-value products, or low-value products and this division is inevitably tied to product price, margin values and final product characteristics. The first group comprises mostly complex molecules, such as biopharmaceuticals, that are usually produced at relatively lower volumes and attract much commercial interest (Budzianowski 2017). High-value products mostly cannot be produced at a very large scale from raw biomass, due to sophisticated conversion routes and the bioprocesses employed, necessitated by required product high quality and purity. For example, an injectable biopharmaceutical, such as a monoclonal antibody, cannot be wrongly folded or contain trace impurities, as otherwise, the life of a patient would be at risk (Kelley 2009). In comparison, low-value products are usually produced at high volumes and do not require as sophisticated platforms as high-value substances or as many process steps.

The earlier considerations suggest that indigo dye should be considered rather as a lower value bio-commodity—the price of a pigment cannot compare with the price of high-value products, such as medicines and biopharmaceuticals, while very large quantities of indigo are required on a global scale (See Introduction). The purification process for indigo biosynthesis is thus not expected as extensive, and the small-scale experiments, relevant to this reaction, back this hypothesis. Most indigo-related research studies and papers were performed on a bench-scale, and they harnessed only centrifugation, extraction, and a single or repetitive wash step with plain distilled water (Han et al. 2011, 2008; Ensley et al. 1983). As no large- or industrial-scale of recombinant indigo is established, no direct comparison was possible, and the later proposed flowsheets should provide new, innovative perspectives.

To increase the relevance of the bioprocess considerations, the successfully implemented DSP processing for other low-value bio-commodities was analysed. For one of the globally most important bio-products, bioethanol, the purification often comprises just 3–4 operations—filtration, one- or two-step distillation process and passage through a molecular sieve (Muhammad and Rosentrater 2020). For a different representant of bio-commodities of a similar class, lactic acid, the DSP bioseparation has five stages: clarification (ultrafiltration), two-step ion exchange (cation exchange resin and anion-exchange resin) and two-step concentration (reverse osmosis and evaporation) (González et al. 2007).

#### Scale-up considerations

Biological platforms and bioprocesses are difficult to scale up from small-scale operations to larger handling volumes due to the inherent nature of biological entities, including their volatility and robust responsiveness to circumstantial conditions (Ratcliffe et al. 2011). While scaling up is usually necessary to obtain industrially relevant and commercially attractive product outputs, it is challenging. Maintaining constant process parameters across scales is difficult, as well as effectively optimising all major phenomena vital for the host, such as nutrient and oxygen delivery or mixing, while avoiding substrates’ gradients as the scale increases (Carpio 2020; Xia et al. 2015).

Apart from engineering the host strain features and optimizing medium composition, pH and temperature, the key factors to consider are mass transfer and hydrodynamics. If incorrectly addressed, these can impose severe physiological limitations, affecting host productivity, product quality and purity (Wang et al. 2015). Effectively optimized fermentation reduces the burden on subsequent DSP operations, that otherwise are mostly increasingly expensive, and may strongly impact process economics and feasibility (Crater and Lievense 2018).

Process parameters that must be intentionally controlled include oxygen transfer rate (OTR), oxygen mass coefficient (kLa), volumetric power consumption (P/V) and mixing characteristics, such as impeller speed, mixing time and varied aspects of vessel geometry (Narayanan et al. 2020; Xia et al. 2015). For more information about varied process parameters, see Carpio (2020) and Crater and Lievense (2018). Although there is no universal scale-up technique for bioprocesses, commonly it relies on keeping chosen of some of the mentioned operating variables constant (Wang et al. 2014). With the increasing emergence of high-throughput screening and scale-down technologies, these methodologies are often harnessed to improve bioprocess development. The most desired process parameters are nowadays commonly established via the coupling of experimental and digital approaches and modelling simulations (Narayanan et al. 2020). Before real-life, practical scale-up, such investigations should be performed for indigo biosynthesis as well to facilitate achieving reliably and repeatedly the best possible process outputs.

### Proposed process flowsheets

#### Final formulation—what characteristics should the final indigo product have?

As mentioned above, the main application for manufactured indigo is for the dyeing industry, especially for dyeing denim fabrics (see Introduction). The relevant industrial product specifications, quality standards and suitable testing methods for dyes and pigments are stipulated by the International Organization for Standardization (ISO), such as ISO/TC 256: *Pigments, dyestuffs, and extenders* (ISO 2022). The exemplary evaluated features are presented in Table 1. Many standards and methodologies have been developed in collaboration with the Ecological and Toxicological Association of Dyes and Organic Pigments (ETAD), an international organization representing colourant-producing companies (ETAD 2022; ISO 2022; Park 2013; Park and Shore 2007). However, often, the manufacturers themselves develop a specific set of in-house methods, while ETAD’s recommendations are rather often readily adopted by dye users, such as the dyeing plants (Park and Shore 2007).

**Table 1:** Comparison of the yield and purity attributes of chemical and biological platforms for indigo synthesis, and a representative summary of key dye product features, that suppliers commonly test for dye standardization

Process Attributes		
	Chemical Platforms	Biological Platforms
Process yield	Difficult to reliably assess; industrial performance of multi-step Pfleger–Heumann indigo synthesis processes is kept as trade secrets (“Chemical synthesis of indigo” section)	Good titers often range 0.3 mg/L–1 g/L, with 18 g/L reported as highest (“Upstream processing (USP) considerations” section)
Product purity	From 96 to 97% for standard processes, to > 99% if additional purification steps would be employed (Kolhaupt et al. 1991; Kolhaupt and Bergmann 1993)	Small-scale studies suggest a potential to match the purity of commercial synthetic indigo (O'Connor et al. 1997). For example, Dai et al. (2019) biologically synthesized indigo of 96% purity, while 94–99% was obtained by Lončar et al. (2019)
Chosen exemplary key dye product features		
Color shade and levelness, fastness, solubility in water, solution stability, dispersion properties, tinting strength and organic impurities content (ISO 2022; Jasper and Günay 2014; Motschi 2003; Park 2013; Park and Shore 2007)		

While the process attributes of yield and purity for biological pathways appear promising and several studies practically tested microbial indigoid dyes for fabric colouration, such as Hsu et al. (2018), Lee et al. (2021), Namgung et al. (2019) or Ullrich et al. (2021), there is a gap in comparative analytical assessment with performance to current industry standard, the synthetic indigo. In comparison, more studies that evaluate synthetic indigo alongside plant-based dye are available (Choi 2020; Paul et al. 2021; Lohtander et al. 2021; Vandenabeele and Moens 2003). This is possibly because both of these products are already prevalent in the industry, while as described, the biological indigo has been investigated rather on a small-scale within academia (“Biological production of indigo” section). Thus, future studies should experimentally examine the commonly evaluated standardized properties of bioprocess-derived indigo. To examine the commercial attractiveness of bioindigo, these values must be compared with the levels obtained with the current industry standard, the synthetically produced dye.

With regard to prevalent product formulation, most manufacturing plants had been providing indigo as a pure dry powder, which has a density above 1 g/cm^3^, and this is still the standard product formulation of this dye (Berger and Sicker 2009). For example, the density of 95% pure indigo is 1.35 g/cm^3^ at 20 °C (Sigma-Aldrich 2022). Synthetic indigo is said to contain only trace chemical impurities in its final formulated pure form (Shi et al. 2021) and has a purity of 94–100% (Wenner and Forkin 2017). After being produced, those products are then shipped to the dyeing unit, which then must reduce the powder pigment, often by chemical methods and the addition of strong reducing agents. This reformulation enables dissolving of the indigo and facilitates performing the procedure of dyeing cotton fabric or yarn (Paul et al. 2021).

However, since its introduction in 1993, there is a growing trend toward the use of pre-reduced indigo products within the textile industry. The shift is claimed currently as very strong in most markets, excluding China (Blueconnection 2019). The pre-reduced indigo is usually a paste or a liquid solution and with its use lesser amount of chemical agents are needed during dyeing, lowering the toxicity of wastewater as well (Blackburn et al. 2009). Relevant tests performed by a major indigo supplier, DyStar, showed a ninefold reduction of sulphites level in the wastewater from 27,084 mg/L to 3,250 mg/mL, when pre-reduced indigo was used instead of traditional pigment (Warren 2020).

The currently available pre-reduced indigo solutions, usually contain 10–50% indigo, water and a reductant (Keegan 2021). For example, DyStar Indigo Vat 40% solution contains 40% indigo, < 2% sodium hydroxide (caustic soda), < 3% potassium hydroxide, and ~ 35% water, while the density of such product is 1224–1232 g/cm^3^ at 20 °C (DyStar 2009; Table 2.). After being produced, the ready pre-reduced indigo solution is then shipped to the dyeing facility, similar to the powder indigo products (Ullrich et al. 2021). However, due to the liquid formulation, the pre-reduced solution does not require the inconvenient dissolving with reductants to be performed by the dyeing unit, a step which must be performed if the powder indigo is used.

**Table 2:** Values that are important for the indigo demand for a large-scale denim dyeing unit. These parameters are later used to assess the required plant capacities that could enable a commercially attractive biological indigo platform

Values important for indigo demand for a conventional large-scale denim dyeing unit		
Amount of annually dyed yarn	36 million metres	A value reported for an industrial Pakistani dyeing unit (Meraj et al. 2016)
Liquid indigo consumption for yarn dyeing	35 g per metre yarn	A value sourced from indigo rope dyeing methodology (Meraj et al. 2016). It is estimated that 95% of denim fabric is produced by this technique, or slasher dyeing (Textiletuts 2021) Within the dyeing industry, indigo may be used to dye the cotton thread, and the established dyeing are rope- and slasher-dyeing and loop dyeing being the third option. Alternatively, finished garments it could be directly applied to dyed formed garments; however, this is not as common. The following text assumes that the indigo produced with the biological platform is used for rope dyeing of the cotton yarn, as this is a well-established established processing option (Paul et al. 2021)
% of indigo in liquid indigo solution	40%	Concentrates contain 10–50% indigo pigment (Graham Keegan 2021). An exemplary commercially available product is, a pre-reduced liquid indigo, that contains: 40% indigo, < 2% sodium hydroxide (caustic soda), < 3% potassium hydroxide, and ~ 35% water (DyStar 2009, 2021a)
Annual indigo supply needed by a large-scale plant	504 t which would dye 200,000 pairs of adult jeans	Equation: (dyed yarn metres/year) × (liquid indigo consumption/metre yarn) × (indigo % in liquid solution)

It is relevant to mention that in recent years there is also a new trend toward investigating the creation of dyed fabric that relies on biological in situ dyeing, instead of using a separate dyestuff product. The dye synthesis is directly followed by fabric dyeing in the same space, merging into one step the creation and disposition of the pigment. The process could rely on growing bacteria directly on a textile and secretion by them of the dye directly on the fabric (Karana et al. 2020). Potentially, the application of purified bacterial supernatant on the material could be investigated as well. In situ platforms could reduce many steps, such as purification, set up of a separate dyeing process or transportation, and bring energy and resource savings (Ullrich et al. 2021).

**Fig. 10:**
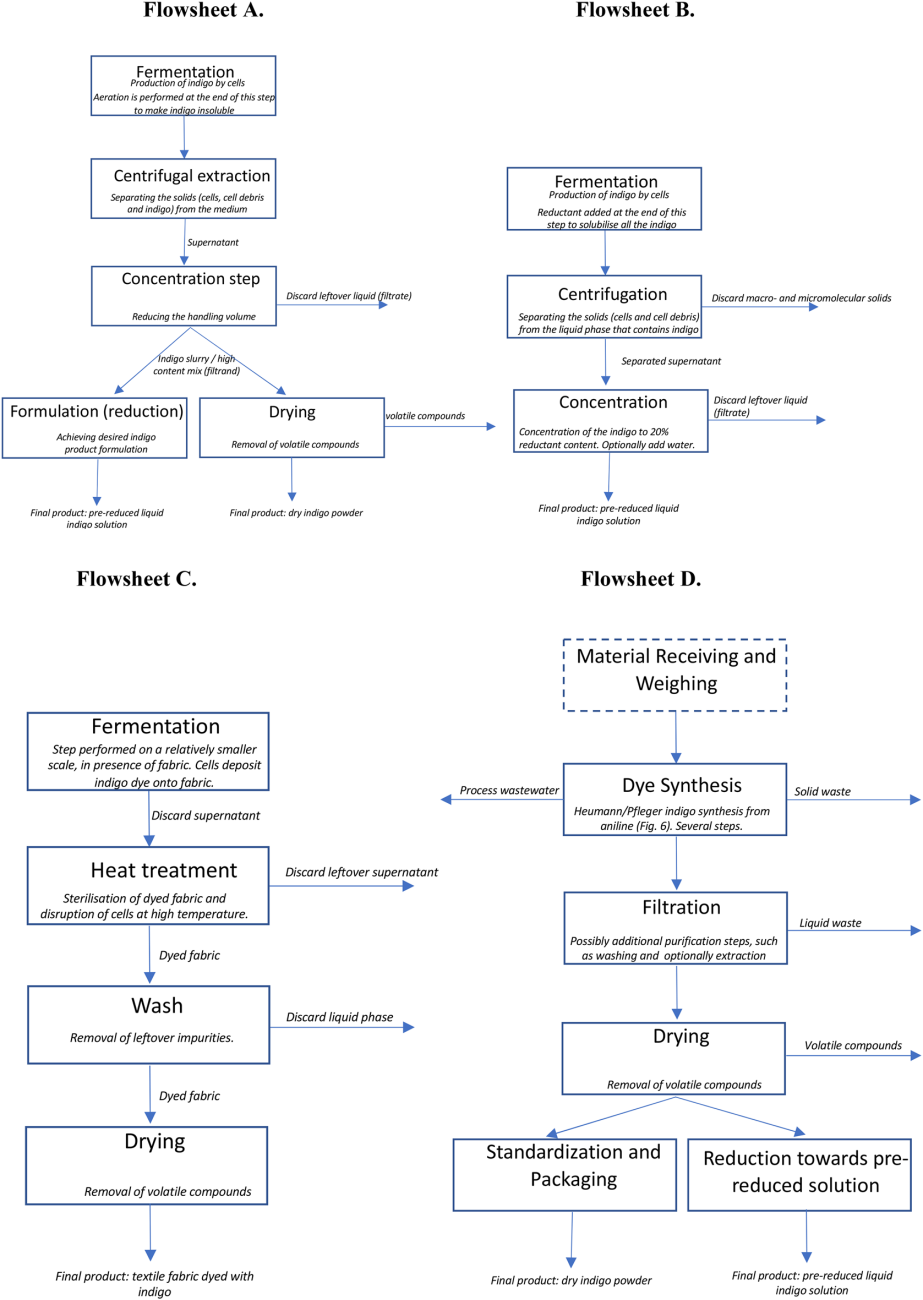
Proposed process flowsheets, that are relevant for the production of commercial indigo dye products. **Flowsheet A–C**. represent flowsheets toward indigo derived via a biological platform, while for **Flowsheet D**. a chemical platform is assumed. **Flowsheet A** The flowsheet that is predicted for the considering platform. It has been assumed that indigo is in the medium after the fermentation, and no cell disruption has to take place. The process relies on the precipitation of indigo after the upstream chemical reaction. Depending on the chosen final step, this bioprocess could produce 2 different formulations of the product; either a powder indigo, or a pre-reduced dye solution. The normal writing represents the name of the considered step, while the *cursive*—the aim of the single flowsheet step or information about discarded or saved feed content. **Flowsheet B** The flowsheet is for the preparation of indigo by solubilization after the upstream chemical reaction. The process output is a pre-reduced solution only. It has been assumed that the indigo diffuses into the medium after production by cells. **Flowsheet C** This scenario represents in situ dyeing—the indigo is secreted onto the fabric. The final product is the dyed fabric. **Flowsheet D** is a comparative diagram of current steps for the production of synthetic indigo by a conventional chemical manufacturing platform, that has been sourced from Paul et al. (2021)

However, the in situ platforms that would be currently practically possible could operate only on much smaller scales, than the conventional industrial-case dyeing platforms, which have to be able to dye thousands of garments or yarn meters annually (Ullrich et al. 2021; Table 1). Thus, it may be difficult to commercially implement in situ platforms in the close future. For such a case the current dyeing system would need to be disrupted, and shift from single industrial-scale plants to multiple smaller scale capacities for the production of dyed fabric. Thus, this paper focuses on more traditional bioprocess, which would provide indigo products that could be directly used in the current denim dyeing system, as realistically that would be easier and more probable to implement. Although a more detailed review of in situ dyeing is beyond the scope of this research, a potential process flowsheet for such a platform has been included to aid the understanding of the main differences between in situ production and more conventional cases (Fig. 10).

#### The choice of the indigo synthesis reaction host and product location after the reaction

The recombinant indigo biosynthesis has been successfully investigated for different bacterial organisms and theoretically many different microbes could be used as a host organism for a fermentation platform (Ma et al. 2018; (“Upstream processing (USP) considerations” section). For the following sections, *E. coli* has been assumed as a host to enable more detailed process considerations. This choice is justified by the fact that many crucial experiments, relevant to indigo bioproduction, were conducted in this host (Murdock et al. 1993; Berry et al. 2002; Han et al. 2011; Hsu et al. 2018). *E. coli* also offers many general advantages such as well-understood physiology and being an established host for the production of metabolic products (Nakamura and Whited 2003). Use of this bacterium would also have benefits which are very specific and relevant to indigo bioconversion, such as the documented achievement of high-flux tryptophan production (Berry et al. 2002) and native possession of the important indigo metabolic pathway enzyme, TnaA (tryptophanase) (Lee and Lee 2010).

If *E. coli* is considered as a whole-cell host, the previously reported experiments did not uniformly and clearly state if the dye substance is present in one specific location after the indigo synthesis—if it is intra- or extracellular, or would be dispersed between medium and inside of the cell in a certain ratio. Most studies focused on the purification of indigo that is produced into the medium. Berry et al. (2002) stated that all biosynthesized indigo is extracellular, while Hsu et al. (2018) considered that although indoxyl is localised within the cell, the glucose derivative of indoxyl, a glucoside indican, was present in the medium as well. When desired, glucosidase was introduced into the system, and after the removal of glucose moiety, the indigo substance was formed after exposure of the system to air.

Moreover, in general, the final step of indigo production via biological pathways is the transformation of indoxyl into indigo in an oxidising environment, for example, during exposure to air or molecular oxygen (Weyler et al. 2001). Such conditions would be found outside the cell mainly, as the extracellular environment is considered as oxidising, while intracellular space is seen as rather highly reducing (Ottaviano et al. 2008). In an Amgen patent, Ensley et al. (1983) mentions that blue indigo crystals are produced by an exoenzyme, which denotes an extracellular biocatalyst, although he states that the indigo frequently crystallises in both, in the medium and in the cells. He suggests that the compound may be, therefore, isolated from the microorganisms and/or from the surrounding culture medium. There is no record of early cell death due to the formation of dye substance crystals—it may be hypothesized that this is not a concern at least for a bioprocess that does not exceed the yield of 18 g/L, that has been achieved without observance of such case by Berry et al. (2002).

The information summarized in this sub-section suggest that possibly there is no active transport of the indigo within the cell and the dye substance could be likely evenly distributed between the intracellular environment, periplasm, and extracellular media broth. Further considerations are pursued under this hypothesis, although future experiments should be conducted to test if this assumption is correct.

*E. coli* are small-sized bacteria that grow in moderate density (Charov and Burkart 2020) and it could be assumed that for a bioprocess the volume of media is much larger, than the volume of cells. Thus, likely the amount of indigo in the extracellular broth would be larger, than within the cells. This could justify why many previously mentioned studies focused on the indigo that was present extracellularly in the medium. Still, the density of most cell types that grow in the suspension may vary, and this includes *E. coli*. To assess the correctness of the statement that the volume of media is much larger than the volume of *E. coli* cells, the Optical Density (OD) values could be used in future to estimate what is the ratio of the total volume taken up by the cells to the volume taken by the medium surrounding them. If further studies reveal that intracellular indigo is formed, then it should be assessed if its recovery would be cost-effective. The disruption of cells requires additional unit operations, and thus time and financial costs, than extracellular separations, what might not be economically worthwhile.

The following process considerations thus assume that only extracellular indigo is recovered and purified, and no cell disruption takes place.

#### The parameters that are relevant for plant capacity considerations

The establishment of the fermenter and plant capacity is a key decision, as this influences the unit operations of the DSP, which changes depending on the magnitude of the product to be processed (Doran 1995). This sub-section aims to briefly discuss relevant aspects that could influence this choice of a biological platform for indigo synthesis and provide the rationale for the pursued scenario.

Many fabric dyeing facilities operate on a very large scale, for reasons such as beneficial economies of scale and high consumer demand for dyed commodities and clothes (Paul 2015). The denim-related plants are no exception—for example, a Chinese denim mill Advanced Denim claims to produce around 40 million meters of dyed yarn per year (Appendix; The Sustainable Angle, 2019). A study by Meraj et al. (2016) also presented similar data from a Pakistan-based denim mill, where the output was 36 million yarn meters per year and at least 40 facilities of such scale function in this country.

A shift in the manufacturing platform is a complex process and has a high opportunity cost—would require significant time and financial resources that otherwise could be devoted to a different project or activity (Chit et al. 2015). If microbial fermentation could not provide industrially significant indigo volumes, it is unlikely that large suppliers would implement such platforms for mass production, even when considering sustainability aspects. This provided the rationale to investigate projections of a biological platform for indigo synthesis that would harness an industrial-scale bioreactor. The annually needed indigo supply for just a single dyer, is very large, 504 tonnes (Table 2). Few commodities are produced at such a scale via fermentation and most are cheap, lower value substances, such as bioethanol, amino acids and vitamins for animal feed (Budzianowski 2017). For outputs of 1000 s tonnes/year, reaction yields may reach 30–40 g/L—an exemplary compound is citric acid (Rywińska and Rymowicz 2010). Simultaneously, the single volume of employed reactors oscillates around 50–250 m^3^, usually closer to 200–250 m^3^ (Eggeling and Bott 2015; Harrison et al. 2015; Meyer et al. 2017). An important decision for the plant design is the choice between single or multiple fermenters. The increase in fermenter number should increase the batch number and improve (increase) DSP utilization, although a multiple-parallel bioreactor platform necessitates a complex control system.

The handling volume of the individual fermenters would likely fall in the range of 100–1000 m^3^ which is the current standard in industrial biotechnology, although the upper-end sizes are more characteristic for the food and feed industry (Li et al. 2020).

In the current scenario, a multiple bioreactor facility is exploited, as this enabled investigating if the biological platform could provide a supply that would be relevant to industrial-scale indigo dye consumers. However, it is worth mentioning that as a practical variable, with available investment the plant size is easier to manipulate than some process attributes, which are subject to biological mechanisms. For example, for volume increase, an additional, spare reactor may be added during the initial design.

#### Product- and process-related impurities—relevance for suitable process flowsheet

The bioprocess flowsheets should provide a rough idea of a possible framework for the indigo synthesis. The chosen units should facilitate removing product-related impurities, such as indigoid derivatives formed due to incorrect hydroxylation (Berry et al. 2002), and process-related, such as biomass leftovers (Fig. 10; Bracewell and Smales 2013).

For product-related impurities, the aim should be to limit their production in advance, before the commencement of fermentation, especially via genetically modifying the host strain and optimising the upstream processing. During metabolic processes toward indigo synthesis, different by-products may be formed, depending on the chosen metabolic route (“Biological production of indigo” and “Upstream processing (USP) considerations” sections)

As a small molecule with a simple structure, indigo has rather low chances of incorrect formation of side-products, such as isatin or other monomeric or dimeric compounds (Gillam and Guengerich 2001). An exception is its structural isomer, indirubin, a common undesired side-product for indigo synthesis, that provides an unwanted reddish tone to the solution (Berry et al. 2002; Gillam and Guengerich 2001). While most biological pathways generally produce indigo as the main product and indirubin as a side-product, bench-top chemistry methodologies, where indirubin is not formed have been reported, especially under conditions of reduced temperature (Shriver et al. 2022). Indirubin has potentially attractive medical applications, and approaches, where its synthesis exceeded that of indigo have been achieved. However, in the context of indigo production, research efforts to reduce indirubin in the final products’ mixture, are greatly needed (Ma et al. 2018). Many studies that experimentally examined the post-reaction mixture content found prevalently both indigo and indirubin, such as Ameria et al. (2015), Berry et al. (2002), Cairo (2015), Catucci et al. (2022), Jin et al. (2022) and Yin et al. (2021). Only O'Connor et al. (1997) reported no indirubin content when a styrene monooxygenase system was used. Apart from this study, little explicit information is available in the literature if and which enzymes can produce indigo solely, with no side production of indirubin. It is possible that enzyme and strain engineering and fine-tuning of reaction conditions could substantially lower the indirubin content. For example, it was observed the faster indole oxidation occurs, the more the final indigo content rises (Gillam and Guengerich 2001). Another promising example is that manipulating only the reaction conditions had a similar effect in a transformed *E.coli* expressing a P450 enzyme (Cairo 2015).

Another approach was investigated by Berry et al. (2002), who designed a modified strain that contained a high level of activity of isatin hydrolase, an enzyme that converts indirubin to isatin acid, a compound that is not an indirubin precursor. This genetic modification did not influence indigo production via fermentation, but the indirubin content in the final product fell by between 50 and 80%. The large-scale dyeing trial that used obtained bioindigo had a quality equivalent to one that used synthetic indigo, providing a good example of how a correct USP modification may improve the biological indigo synthesis.

The final purification protocol relies then heavily on the upstream design and decisions such as which bioconversion route would be pursued, and the constitution of media and nutrients provided to the system. The removal of process-related impurities is relying largely on downstream processing, meaning the unit operations after the fermentation step.

#### The constructed bioprocess flowsheets for indigo production

Figure 10 presents 2 different flowsheets, constructed for indigo biosynthesis and purification. An important aspect is that at the beginning of processing after fermentation, indigo is in solid form, due to its insolubility in aqueous solutions, while its molecules are relatively small, and a single one weighs 262 Da (Głowacki et al. 2012). The proposed Flowsheets A. and B. are compatible with the supply chain for potential biological indigo platforms, which was presented in Fig. 7 in “Overview of technically feasible bioprocesses and their contextual evaluation” section.

The proposed Flowsheet A. was initially designed to produce a powder pigment, as it is a more versatile product—this form could be sold not only to commercial dyeing units, but also very conveniently to other customer segments, such as artisans and artists (Centre for Global Equity 2021; Paul et al. 2021). If the commercialisation of indigo biosynthesis would necessitate only a gradual expansion of production volume, the initial target buyers could originate from these groups as well. A relevant fact is that there is nowadays a strong trend between fashion and art consumers to shift preferences toward more sustainable wearable goods, even in exchange for relatively higher prices (Gorman 2020). Thus, the biological indigo may benefit from this social observation and movement. Interestingly, with just a single-step substitution of the final formulation operation, a pre-reduced product could be obtained, as illustrated on flowsheet A as well (Fig. 10). Such a process could be interesting to commercial companies as it offers a possibility of product diversification, and thus expanding the pool of potential clients. For the reduction step, from the known employed operations, catalytic hydrogenation or electrochemical reduction would be more sustainable choices, although the direct chemical methods are prevalent, mainly the addition of alkali (Blackburn et al. 2009). Thus, a single facility could potentially decide to produce both, powdered indigo, and a pre-reduced indigo solution.

Although there is potentially no need for fully different processing for powder indigo product and a pre-reduced solution, Flowsheet B. has been proposed to visualize a case that focuses solely on pre-reduced indigo. It harnesses the fact that the final product contains reductants, such as sodium hydroxide, which could be used to extract the indigo from the medium as well, as it shifts the indigo into its soluble leuco form (“Physicochemical properties of indigo” section) In real life, the potentially shorter Flowsheet B for pre-reduced indigo solution may be more cost-effective than Flowsheet A., while the trend to replace the indigo powder with the pre-reduced solution is steadily growing within the denim dyeing industry (Blackburn et al. 2009; Paul et al. 2021). This could be investigated in future, for example, via the Life Cycle Assessment (LCA) or relevant techno-economic evaluation.

Flowsheet A. is the main flowsheet, that is being proposed for a later designed biological platform for the biosynthesis of indigo via harnessing microorganisms. After an upstream fermentation reaction, it consists of 3 DSP steps, where there are 2 possible choices for the final formulation step, depending on the desired final indigo product form. This Flowsheet A., and notably its pathway toward the more common and widespread powdered product, has also been chosen as the base for the potential biological indigo output calculations, that will follow in next “Platform output analysis” section. Interestingly, if a platform would yield only the liquid dye solution, a shorter two-step DSP could be potentially employed, and Flowsheet B. represents this scenario. Flowsheet C. is presented to provide yet another perspective and visualize the trend toward the in situ dyeing processes, which may be highly attractive for future solutions, although now due to technological and practical limitations may be not yet fully developed for employment on an industrial scale, as discussed above. The final Flowsheet D. presents the process, that based on the available literature is most likely employed in the current chemical platforms. Its inclusion enables a comparison of the current chemical-based production with the proposed bioprocesses from Flowsheets A–C.

This concise length of the **Flowsheet A**. DSP is assessed as a likely procedure, as for most reported relevant experiments, the purification comprised only 2–3 steps. For example, Hsu et al. (2018) laboratory experiment and Han et al. (2011) pilot fermentation trial both commenced with centrifugation after the upstream reaction, to separate the cells and indigo—which is solidified in the non-polar medium—from the liquid. The latter study explicitly mentioned that the pellets collected at this point were dark blue, which indicates that the indigo was likely in its insoluble form, as the soluble leucoindigo is pale yellow (“Physicochemical properties of indigo” section, Fig. 1).

Next, both studies harnessed indigo’s solubility in organic solvent—dimethyl sulfoxide (DMSO). The first study then reconstituted the pellets, which were collected after initial separations, in DMSO for second centrifugation, while the latter study included a wash step with distilled water and drying after first centrifugation, and cells resuspension in DMSO (or DMF) and further sonication for quantification purposes. Hsu et al. (2018) experiment included dyeing a cotton scarf, although not directly with obtained indigo but with indican, a glycosylated indoxyl derivative, thus this dyeing procedure was assessed as not relevant enough to include it for these flowsheet-related considerations.

However, with a significant increase in working volume, often the complexity of bioprocesses rises (Cossar 2019)—while this section is largely theoretical, in real-life one has to prepare for different cases. Thus, it could be possible that 1 or 2 additional DSP steps would be needed to achieve sufficient purification of biologically produced indigo, although this is not predicted as highly likely. The following text will firstly explain the major unit operations that have been proposed for Flowsheet A and their rationale. Then, the additional Flowsheet B. and C. will be discussed, and Flowsheet D., which represents the chemical indigo synthesis and enables comparison with the current standard of synthetic indigo manufacture.

#### Isolation and primary recovery

For both, Flowsheet A. and B., once the fermentation step for indigo production is finished, a suitable DSP operation should commence with a primary recovery unit to collect the solidified indigo from the liquid media phase, where the product is assumed to remain after its formation by bacteria, basing on “Upstream processing (USP) considerations” section. considerations. An important difference is the starting form of the substance at the very beginning of the first purification steps and the actions involved to achieve it. The former scenario harnesses the insolubility of indigo upon oxidation, which is proposed to happen via aeration, while on the contrary, the latter Flowsheet relies on fully solubilizing the indigo via addition of reducing agents. It could be argued that the former operation is not entirely environmentally friendly, although this does not hold, as these reagents would not form the waste stream from this bioprocess, as they would remain in the final pre-reduced indigo solution, as the containment of reducing agents is necessitated by the content of the common real-life products of that type (Table 2.).

No operation devoted purely to cell disruption has been predicted, as multiple scientific studies have focused on extracellular indigo and the designed bioprocess follows this approach as well.

In the context of the above-mentioned primary operation, in line with process engineering principles sourced from Harrison et al. (2015), firstly the most plentiful and easiest-to-remove unwanted feed component should be targeted. For the considered biological platform, the major large-sized solid components of the fermentation feed are the *E. coli* cells themselves and the cell debris. For Flowsheet A., a centrifugal liquid–liquid extraction, known simply as centrifugal extraction, is suggested for this purpose, while for Flowsheet B this could be a more standard centrifugation step. Both units harness the sedimentation of particles, which is accelerated by the application of force (Stephen 2018), although the former combines this with an extraction operation by the involvement of 2 immiscible liquids. This unit operation is identified as a desirable process intensification technique and has been employed for many commercial applications. Examples include the production of antibiotics, i.e. penicillin (Likidis and Schügerl 1987), amino acids, i.e. phenylalanine (Rüffer et al. 2004) or coconut oil (Nour et al. 2009; Nurah et al. 2017). Centrifugal extraction has the advantages of shorter processing time, capital savings and often increased step effectiveness, although may be tied to the increased initial investment (Hamamah and Grützner 2022). There are many available centrifugal extractors (CE), with the main division into a differential or stagewise extractors, where the former exercises continuous operation equivalent to several unit stages, while for the latter each mixing and separation of 2 phases is discrete (Schügerl 2013). For example, many miscellaneous applications rely on annular centrifugal extractors, which are reported as small and efficient, including the pharmaceutical industry or biofuels (Meikrantz et al. 2002; McFarlane et al. 2010). Alternatively, instead of the centrifugal extraction a more standard combination of 2 individual steps, a separate centrifugation and extraction, may be utilized, and these steps are described in later paragraphs.

Flowsheet B. provides a slightly different case for considerations, as it has been assumed that the indigo is made soluble prior to primary recovery. Thus, while the cell solids would become pelleted and be removed upon centrifugation, the desired indigo would stay in the leftover liquid feed; therefore, there should be no need for the extraction component.

As mentioned, in industrial biology facilities employing bioreactors between 100,000 and 250,000 L, and up to 500,000 L, at the upstream stages is common (Humbird 2015; Meyer et al. 2017; Li et al. 2020). In the later proposed commercial platform scenario in “Platform output analysis” section, the volume of the proposed fermenter employed for indigo biosynthesis, is predicted as large as well (250,000 L; Table 3.). Thus, a centrifuge or the centrifugal extractor would likely be operated in a continuous mode, where solids can be discharged when desired, without disrupting the feed input (Abduh et al. 2013; Harrison et al. 2015; Angelis et al. 2021). For classical centrifugation, the suggested equipment could be a disc-stack centrifuge, as it enables such a mode, while offering high sedimentation areas and known scale-up procedures (Harrison et al. 2015; Hagel et al. 2008).

**Table 3:** Assumptions for the design of a large-scale indigo bioprocess with their values and rationale. It is assumed that the platform relies on the bioprocess sequence of Flowsheet A. (Fig. 10). For detailed calculations see Additional file 1

Input assumption		
Parameter (unit)	Value	Rationale
Values crucial for biological production		
Titre	18 g/L	The indigo synthesis titre depends heavily on the upstream choices, such as the choice of bacterial strain, for example, one with available or not high-flux tryptophan pathway modifications, and the enzyme chosen for indigo bioconversion. From small-scale studies, good titres could be assessed as between 0.3 g/L to 18 g/L, when the tryptophan pathway modifications are, respectively, not harnessed (in multiple studies) and harnessed (by Berry et al. 2002) (See “Upstream processing (USP) considerations” section) For simplicity, the titer figure is assumed as pure indigo, and this value already accounts for the incorrectly formed product, such as trace amounts of indirubin (Berry et al. 2002)
Fermenter Size	250,000 L (250 m^3^)	In the industrial biology sector, reactors between 100,000 and 250,000 L are common (Meyer et al. 2017; Li et al. 2020). Exemplary applications incl. the production of commodity antibiotics, such as erythromycin A (Zou et al. 2012), butanol (Lee et al. 2008); biofuels like bioethanol (Humbird et al. 2015), citric acid, lactic acid, pigments, such as astaxanthin (Panis and Carreon 2016), amino acids such as glutamate or l-lysine, for example, the latter being produced in 500 m^3^ bioreactors (Eggeling and Bott 2015) For simplicity, the fermenter size is considered here as the working volume
Fermenter Number	2	Industrial setup of low-value commodities usually harnesses several bioreactors to achieve significant capacities and using several fermenters is common (Doran 1995; Meyer et al. 2017)
Purification (DSP) yield (%)	75%	DSP usually comprises several unit operations, as can be seen on flowsheets proposed for indigo purification as well (Fig. 10). The overall DSP yield is dictated by the number of unit operations and their individual yields. For 3 DSP steps, the overall yield could often oscillate around 60–90% (NBC2, 2016), and 75% has been assumed for simplification
Facility capacity (L)	500,000 L (500 m^3^)	Equation: (fermenter number) × (single fermenter size). The capacity size was also benchmarked against possible and technically feasible real-life biotechnological plants (Eggeling and Bott 2015; Humbird 2015)
Fermentation time	72 h	Culture time required to achieve earlier assumed 18 g/L yield (Berry et al. 2002)
Batch runs per year (for a single fermenter)	80	The achievable number of batches is influenced by the growth rate of the employed microorganism, as it influences the fermentation duration (Doran 1995) 18 g/L has been achieved with 3-day long *E. coli* fermentation; therefore, 2 batches per week have been assumed as possible. To account time for planned or unexpected downtime, 40 weeks of operation per year has been assumed for the bioprocess plant, giving a total of 80 batches scheduled per annum. A study that presented a different scenario with similar assumption may be seen in Pollock et al. (2013) In real-life the schedule batch is also influenced by the longest step of the whole bioprocess, which for industrial scale may not be fermentation. It may be the DSP, as its steps are typically based on flowrates, marking how much feed volume can be run on a certain equipment beyond which the performance substantially decreases. However, for simplification it is assumed that the DSP would not constrain the batch scheduling, which is not unlikely given that for cheaper biocommodities the DSP is not extensive, as when compared to sophisticated processing of high-value commodities (Budzianowski 2017)
Calculated indigo output from a single fermenter from a single run (after DSP)	3.375 tonnes	Equation: (titre) × (single fermenter size) × (DSP yield)
Calculated annual indigo after-DSP output for values given in this table—for 2 ferementers, each run 80 times	540 tonnes	Equation: (fermenter number) × (output from single fermenter per batch) x (batch runs/year)

Alternatively, filtration could replace centrifugation, although at very large scales the latter is usually preferred (Harrison et al. 2015). Another modification could be the use of different equipment, such as tubular type centrifuge or annular centrifugal extractor, although the former disadvantage of complicated cleaning, where the whole operation has to be stopped to scrape the solids and remove them from the centrifugal bowl (Leung 2007).

If the Flowsheet A scenario would rely on a combination of discrete centrifugation and extraction steps, the impurities remaining in the liquid phase, recovered from centrifugation, are predicted mostly as different soluble process-related compounds, such as trace amounts of salts or proteins, that were secreted by host cells, and the cells themselves. An important and unique difference between these feed components and indigo is its solubility and stability in organic phases and solvents and its insolubility in aqueous media, while most cell- and fermentation-related products are water-soluble. Thus, liquid–liquid extraction (LLE), known also as solvent extraction, is proposed as the next major unit operation. It relies on connecting two immiscible liquids and solute partitioning due to chemical potential. In this step, an organic solvent (a nonpolar substance) would be used to extract indigo from the originally aqueous feed (a polar substance). Most macromolecular impurities should remain after the operation finishes in this latter phase—in the raffinate phase, which would be then discarded, while the product of interest, here indigo, would travel to organic solvent, and this mix would constitute the extract phase.

A suitable piece of equipment for the LLE could be a set of interconnected mixer-settlers—extraction is often conducted in a multistage manner, where for each stage two streams, the aqueous feed and the solvent, are contacted (mixed) and separated before further processing. A multi-stage extraction may provide high recovery yields and is an established step and is commonly used in industrial biotechnology, for example, for the production of antibiotics (Antony et al. 2021) and appears as a relevant choice for indigo extraction. A continuous counter-current cascade extraction mode could be considered, where the raffinate from a single stage, which is the waste stream, is fed into the next as the aqueous phase, while the organic solvent phase is directed in the system in the opposite direction. This solution supports high product recovery and uses less solvent volume more effectively (Münzberg et al. 2018).

A few alternative solutions could also be potentially implemented—for example, a co-current cascade mode, where in each step a fresh solvent is introduced to the system. However, this suggestion may cause an increase in material and equipment costs and in the post-step chemical waste (Pushpavanam and Malengier 2012). LLE could also harness and rely on other differences in characteristics between indigo and remaining impurities, for example, ion-exchange extraction is sometimes employed and could be relevant for indigo purification. Indigo may exist in reduced forms under basic conditions, as the pH increases: the acid leucoindigo, the monophenolate ionic form of leucoalkaline indigo or its biphenolate form, as described thoroughly by Blackburn et al. (2009) or Paul et al. (2021). This phenomenon appears as potentially worth harnessing, although the indigo molecules gain water solubility with reduction, thus the step would have to be designed to still effectively enable passage of this solute into the solvent phase, possibly with a pH gradient.

#### Purification

In the Flowsheet A case, a concentration-focused step should follow to reduce the handling volume and aid the formation of insoluble indigo, which is commercially the standard formulation for this pigment (Blackburn et al. 2009; Blueconnection 2019). Different filtration modes may be used for that purpose, or potentially precipitation step.

For example, a rotary vacuum filtration and rotary drum drying is an established DSP sequence for the purification of citric acid (Dhillon et al. 2013), which is another low-value commodity obtained via fermentation, for which the huge annual production volume exceeds 1.7 million tonnes (Anastassiadis et al. 2008). This filtration type uses a large drum, which is rotating in a liquid and covered with a membrane—a vacuum sucks the feed onto the drum outside, and the product is then recovered with a knife (Tarleton and Wakeman 2007). Alternatively, another filtration type could be used for the purpose of concentration, and a different drying methodology. For example, ultrafiltration may be considered, especially in the cross-flow mode, where the feed passes in parallel to the membrane and cell cake does not accumulate and block the membrane (Nabais and Cardoso 1995; Bhave 1996). However, the rotary vacuum is preferred for slurries and high-solid content streams, which could otherwise clog other filters, what could be a risk while purifying indigo—the knife scrapes the product and allows exposure of fresh membrane. The following drying operation would serve the removal of any unwanted thermally volatile substances, especially that the product recovered from the rotary drum is usually relatively wet (Tarleton and Wakeman 2007). Indigo is quite stable at higher temperatures and can withstand conditions of 50–100 °C (Głowacki et al. 2012), thus many different modes of drying could be potentially used, including spray, fluidized-bed or tray modes (Harrison et al. 2015).

**Fig. 11:**
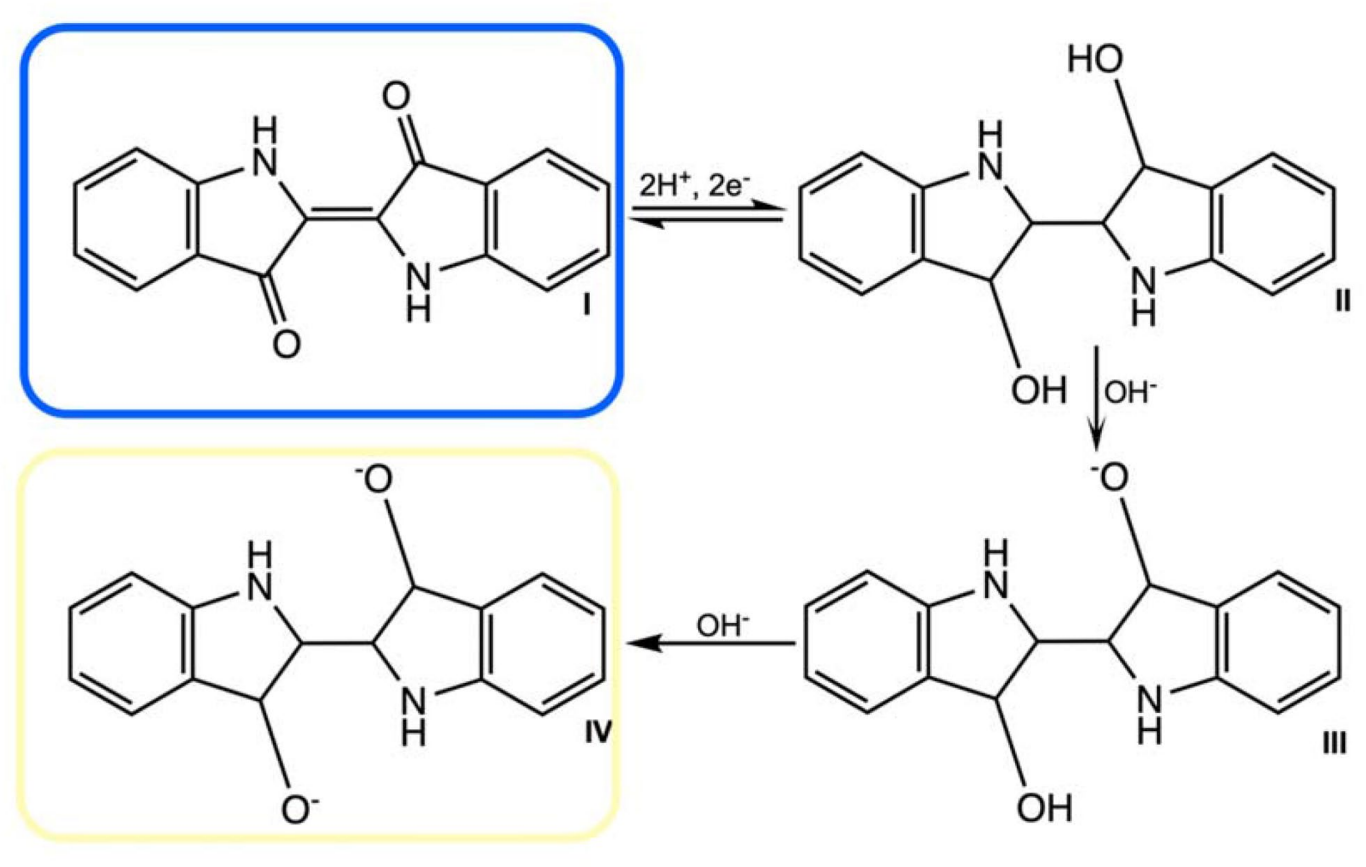
Different indigo forms: I.—insoluble parent pigment form; II.—acid leucoindigo; III.—monophenolate ionic form of alkaline leucoindigo; IV—biphenolate ionic form of alkaline leucoindigo. The drawn rectangles mark the expected colour of the compound

In the different approach with mentioned precipitation, the compound of interest is selectively precipitated via changes in phase environment, such as modification in temperature, ionic strength or pH (Harrison et al. 2015). For indigo, the pH could be harnessed—as mentioned earlier, as the pH increases indigo may exist in different reduced forms under basic conditions (Fig. 11)—or perhaps its insolubility in aqueous mediums.

#### An alternative scenario—Flowsheet C

As mentioned above, Flowsheet C represents a potential bioprocess for a different scenario, where the platform goal is not the production of a dye product, but to yield the finished dyed textile, yarn, or fabric (Colorifix Ltd, 2016). When compared to Flowsheets A and B, the main difference occurs up-front at the upstream reaction setup and its output. The microorganisms do not secrete the desired indigo molecules into the medium, but directly onto a fabric due to their co-culture with it during fermentation. Importantly, in situ process not only takes the burden of the transportation to a separate facility for dyeing and discrete processing of this activity and the dye production, but positively impacts the DSP involved in the main bioprocess. While during USP the dye had already been adsorbed onto the fabric, almost all other undesirable macro- and micromolecular process-related compounds, such as polysaccharides, nucleic acids, and proteins, will be in the liquid medium (Rathore et al. 2018). The current commercial entities, that are developing in situ platforms, such as UK-based Colorifix, employ after the fermentation a heat treatment step to sterilize the fabric from biological and living components. This is then followed by a wash step and drying, after which the finished dyed fabric is ready (Nugent and Ajioka 2016). This purification sequence is not only concise and may be predicted as relatively cheap, but it does not require any toxic chemicals or solvents. Thus, it has the potential of being one of the most environmentally friendly paths to obtain dyed commodities, which is the final goal into which indigo production plugs (Paul et al. 2021). Still, as argued above, this approach has technical limitations and may be difficult to adapt by the industry due to its novelty and requirement of system disruption by moving from large-volume platforms into smaller scale ones.

#### Comparison of bioprocesses with the current chemical processing

Flowsheet D represents the current standard chemical process of indigo production, as opposed to the rest of Flowsheets A–C, which consider biological platforms and do not have yet real-life realization. Their main advantage over Flowsheet D is the removal of the need for using toxic and harmful substances during production, and the decrease of the toxic waste feeds, that contain those and are challenging to handle responsibly (See “Chemical synthesis of indigo” section).

From the context of purification, the number of required steps for chemical processing is similar to the bioprocess-based Flowsheets, even if the former uses much more toxic and strong substances. This is likely caused by the above-mentioned fact, that during the long chain of chemical reactions toward dye synthesis, the by-products are not removed by the manufacturers. Moreover, on a global scale, most of the indigo— ~ 87%—is produced nowadays in low-income countries (Paul et al. 2021). In those regions, the regulations related to use of the toxic chemicals and the handling of following waste streams are not as regulated, burdensome and expensive for the companies, as in the wealthier regions, such as the USA or Europe (“Toxicity issues” section).

The first major process step in the Flowsheet D includes the chemical chain of reactions toward the synthesis of the indigo, which usually follows the earlier described Heumann/Pfleger pathway (See “Chemical synthesis of indigo” section). The main purification operation relies on filtration, although extraction and wash steps may be involved as well (Paul et al. 2021). In the context of the second important product, the pre-reduced dye solution, the drying step is usually performed to a certain extent—the indigo is firstly dried and formed into a powder, and this powder is then taken, exposed to catalytic hydrogenation or strong reducing agents, and dissolved. The chemical process on which the Flowsheet D has been based, may be also seen from another perspective in Fig. 7, where it had been included in the supply chain overview, which was identified as relevant to indigo synthesis.

### Platform output analysis

#### The feasible plant capacities for a biological indigo platform

Many fabric dyeing facilities operate on a very large scale, for reasons such as beneficial economies of scale and high consumer demand for dyed commodities and clothes (Paul 2015). The denim-related plants are no exception—for example, a Chinese denim mill Advanced Denim claims to produce 36.5 million meters of dyed yarn per year (Appendix; The Sustainable Angle 2019). A study by Meraj and Saeeda (2016) also presented similar data from a Pakistan-based denim mill, where the output was 36 million yarn meters per year.

For comparison, it must be then recognized what bioprocess plant capacities, that are employed in real-life, could be predicted as feasible to employ for a platform for indigo biosynthesis. This may then lay the foundation for the biological indigo output calculations, that follow in later sections, and their relevance for stakeholders.

The physical production size of “industrial-scale” fermentation platforms is dependent on the manufactured product type (Doran 1995). The annually needed indigo supply for just a single dyer, is very large, 540 tonnes (Table 2). Few commodities are produced at such a scale via fermentation and most are cheap, lower value substances, such as bioethanol, amino acids and vitamins for animal feed (Budzianowski 2017). These products form the industrial biotechnology sector, and more examples of relevant products may be seen in Table 3. On the other hand, many bio-products are obtained with lower yields, such as 1–10 g/L, and annually much lower quantities (Lodish et al. 2000). For high-value substances, such as biologics, the reactor volumes often oscillate from around 10 000 s litres to 100,000 s litres, although for some products, such as autologous therapeutics, reactors are substantially smaller. Relevant facilities often employ multiple medium-sized bioreactors to increase the volumetric capacities while minimizing the expensiveness of a single batch error (Farid 2007). Still, this significant segment is valued in billions—many monoclonal antibodies on the market reach 0,25-1t/year production outputs (Kelley 2009). However, this is not entirely relevant for indigo biosynthesis, which, due to being a dye and having lower purity requirements, would rather be classified as a low-value biocommodity. Bioprocessing plants, that produce small molecules, as which indigo would be classified as well, often employ several bioreactors, commonly of sizes 150–200 m^3^. Fermenters up to 500 m^3^ may be perceived as commonly existing as well, while the largest practically possible bioreactor is 1000 m^3^ (Garcia-Ochoa and Gomez 2009; Eggeling and Bott 2015; Humbird 2015, 2021; Humbird et al. 2017). For outputs of 1000 s tonnes/year, titres often reach 30–40 g/L, while single reactor volume up to 250 m^3^ (Harrison et al. 2015; Li et al. 2020). For example, Meyer et al. (2017) presented real-life data from antibiotic production, cephalosporin C, that uses 6 fermenters 120 m^3^ each, totalling a 720 m^3^ volume capacity. If a bioprocessing platform involves more than 1 bioreactor, sometimes multiple fermenters would be run in an overlapping manner, and the implications of this approach are discussed below alongside the potential batch scheduling.

In plant facilities, that are devoted to biotechnological commodities, the operation may focus on a single product or facilitate the production of different products—either at the same time, or during separate and distinct time periods (Sofer 2005). For simplicity, the following considerations assume a monoproduct facility.

#### Could the output of the fermentation platform satisfy the current demand for indigo?

The following considerations, available in Table 3., investigate if from the technical perspective, a microbial fermentation process could supply the large indigo quantities that are currently consumed each year by a single dyeing plant (Table 2). Table 3. contains the relevant process parameters and production aspects that were seen as vital to realistically calculate possible platform output, including the justification and rationale for values.

The outputs and economics of process-related engineering analysis may shift, depending on the level of detail of the considerations and available data, and the relevant technological complexity and novelty of the platform as well. A well-established textbook by Harrison et al. (2015) suggests 5 different levels of types of estimates with error percentages between ≤ 10% and ≤ 50%. For the following indigo considerations, which may be categorized as preliminary engineering or project planning estimates, the accuracy of the analysis is predicted as potentially likely to shift by 25–30%. A relevant fact is that in reality, the data relevant to the production of bio-dyes is either made uneasy to access and find, or kept as trade secrets by companies due to commercial reasons.

#### Table 3 parameters description and discussion

With the values assumed in Table 3., the output from a single fermenter (1 × 250,000 L), after product purification, was estimated as ~ 3.375 tonnes. It is a very good output—if during the conventionally used rope dyeing process, dyeing 1 m yarn uses 35 g 40% liquid indigo (Table 3), a single batch would enable dyeing ~ 241,000 m yarn (Additional file 1). The following considerations will reveal the reasoning behind the assumed parameters to show the thought process that yielded the calculated output values. The further sections assess then the real-life context of the anticipated production scale and its relevance for the denim dyeing industry.

A value complex to assume was the bioconversion titre. In the published literature, the highest indigo biosynthesis reaction yield was relatively high 18 g/L. Berry et al. (2002) achieved mentioned yield with engineering for indigo bioconversion an *E. coli* strain, that is used industrially to overproduce tryptophan at 40 g/L. An indole metabolic pathway plasmid was added, and one encoding an indigo-producing oxidoreductase system, NDO (“Upstream processing (USP) considerations” section). Thus, the tryptophan was not accumulating, and the high flux of the pathway was not curtailed and proceeded through to the formation of indigo. Surprisingly, since this experiment, the reported indigo yields, considered as good, were < 1 g/L; however, no later study harnessed tryptophan-overproducing strains. Han et al. (2011) achieved the highest 911 mg/mL yield in a pilot-scale 3000 L batch fermentation with recombinant *E. coli*. The hypothesis is that the scientific community is aware of and probably uses the modifications mentioned above, thus research focuses on purely evaluating the most effective oxidoreductase system for the key pathway step, indole oxidation (“Upstream processing (USP) considerations” section).

In real-life, throughout a bioprocess, some product is lost during processing and DSP yield values account for that, while the titer value already accounts for an ill-formed product, such as undesired indigo’s structural isomer, indirubin (Table 3.). In the considered potential plant for simplicity, the fermenter size is considered as the working volume, thus it must be recognized that the real-life reactor would have to be slightly larger to achieve the anticipated outputs. The fermentation timeframe was assumed 72 h, as with this culture time the assumed 18 g/L titre has been obtained via *E. coli* culturing (Berry et al. 2002).

The fermentation duration is dictated by the growth rate of the employed microorganism, and for a 3-day long process, 2 batches per week may be scheduled (Doran 1995). Many fermentation-related plants are operated 7 days per week due to 2 important reasons. Firstly, the biological entities have limited stability; therefore, between the product processing steps, spare time should be minimised. Second, a facility is inextricably linked to substantial capital investment. Plant usage is planned with the aim to maximise the financial return and optimise operating costs and idle reactor time would likely have a high opportunity cost. The periodical batch number is dictated by the timeframe of the whole bioprocess timeframe, which includes both UPS and DSP activities. Often the DSP may take longer, as there is a certain maximal flowrate that purification-relevant equipment, such as centrifuges or filters, may be used, as otherwise, the step performance could be poor. Moreover, even for certain fermenter sizes, the actual number of regularly scheduled fermenters depends as well on utilities, including chilling and cooling capacities, water and power use. Those aspects are often narrowed down during later engineering design stages, and therefore, the approximate number of the batch and output estimations must be recognized.

Moreover, as it was mentioned above, for a platform that uses multiple bioreactors, their runs may not be set up simultaneously, but in an overlapping manner. Such a set up may support more effective batch scheduling and increase the batch runs per see, but also could make the downstream processing and resource management easier. For example, from multiple bioreactors, a single fermenter would run for 2 or 3 days before starting the next one. If fewer bioreactors are employed in a single run at once, the handling volume of the feed is smaller, and therefore, performing the DSP steps is more convenient. The DSP equipment used could be smaller, while otherwise it would often increase the requirement of the manufacturing facility footprint, and could be either very expensive to purchase, or even be not easily available from suppliers.

Nevertheless, from the above consideration, the value of 80 runs per year, used in the bio-indigo case considerations accounts for possible downtime, as a 40-week case has been assumed for bioprocess plant operation (Pollock et al. 2013).

#### Estimating denim fabric and jeans output

It is challenging to evaluate precisely, how many metres of fabric could be made with a specific amount of dyed yarn or how many pairs of finished jeans. The indigo is usually applied via the dyeing of the cotton thread, and the established dyeing are rope- and slasher-dyeing, with loop dyeing being the third option, which is differentiated by the fact that only a modified process with only a single indigo bath is used, whereas the former pathways need multiple indigo baths. Alternatively, finished garments may be dyed, which is more sustainable and resource-efficient, although less prevalent within industrial plants (Paul et al. 2021). The following text assumes that the indigo produced with the biological platform is used for rope dyeing of the cotton yarn.

The warp yarn used for denim is uniquely processed, when compared to most woven materials. After the rope dyeing step, addressed, for example, by Meraj and Saeeda (2016), the yarn would traditionally go through at least 4 more formulation steps, including re-beaming, slashing, weaving, and finishing, and only then enter finished fabric operations (Cotton 2004). A more detailed description of relevant dyeing operations is available in Blackburn et al. (2009), or Paul et al. (2021).

The fabric weight of various final denim products also differs, as during processing, the used yarns are formed to contain a specific number of warps, resulting in varied yarn thickness, known as ‘yarn count’ (Cottonworks 2021; Cotton 2004). There is no universal standard denim weight, while the type of denim fabric is widely categorised and characterised by its weight in ounces per square yard (oz.). In general, 12–16 oz. denim fabric is considered midweight, and values below or above that range may be described as lightweight and heavyweight, respectively (Denimhunters 2021).

It was uneasy to find the equivalent of yarn metres that form a single pair of adult jeans. If this could be assumed as 180 m of yarn (Coats 2021), then with current Table 3. values, the output of ~ 3.75 tonnes of indigo dye from 2 fermenters (2 × 250,000 L) could dye 1339 pairs of adult jeans (Additional file 1).

To visualise from another perspective, if one assumes that rope-dyed yarn could be used to make a garment such as a sweater, and that a knitted sweater for an adult woman requires around 1400 m yarn (Cubley 2019), with ~ 3.75 tonnes of indigo, the yarn for 172 sweaters could be provided. 1339 pairs of adult jeans or 172 sweaters could be sufficient for the release of a single luxury collection of clothing and represents a good start for commercial considerations.

#### Relevance for the denim industry

After the absorption of indigo, usually, the dyed yarn would be used for manufacturing that produces denim fabric (Schimper et al. 2011). The annual output of the recombinant platform for indigo biosynthesis was calculated as 540 tonnes (Table 3.) and theoretically, this amount should enable dyeing ~ 27,550 sweaters or ~ 214,200 pairs of adult jeans (Additional file 1). This might be seen as a good output quantity of clothing not only for an *haute couture* fashion designer or high-fashion houses, but also for industrial denim clothing providers, that mass-produce garments. The anticipated annual outcome of 540 tonnes would fully satisfy the indigo supply required by a single large-scale dyer, which was calculated as 504 tonnes (Tables 2 and 3). However, even in Pakistan alone, there are at least 40 industrial dyeing units (Meraj et al. 2016) and it is probable that the indigo suppliers have contracts with more than a single dyeing plant or client, probably at least a few. The full alteration or diversification of manufacturing processes is often a high-cost operation, and the relevant industrial stakeholders would still likely, even roughly, assess the opportunity cost of introducing the proposed biological indigo platform before investigating its technicalities in depth.

#### Sensitivity analysis—assessing the most impactful process attributes

The earlier considerations assessed anticipated platform output for the current state of knowledge and values that were reported and appear realistically achievable. However, after platform establishment research activities would likely continue—most bioprocesses are constantly optimised. For example, in 1980/90 s the monoclonal antibodies’ yields were < 1 g/L, while currently 5 × higher values are common, while some reach 10–13 g/L with extended culture duration (Kelley 2009) and research for process effectivity and productivity constantly continues and a 25 g/L titre was achieved at bench scale (Kuczewski et al. 2011).

**Fig. 12:**
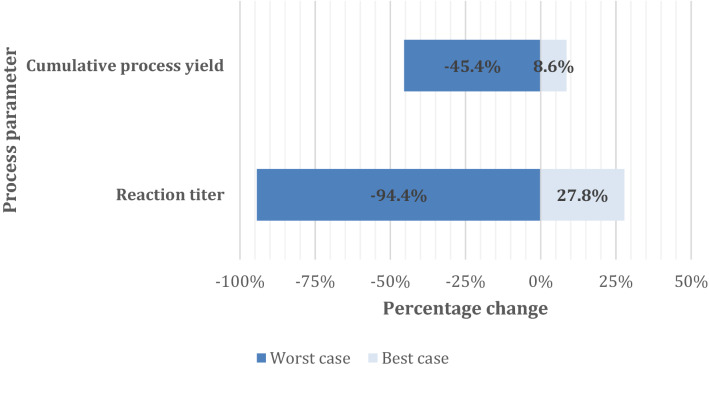
Sensitivity analysis chart, that presents percentage change in the annual output of indigo from a biological platform for scenarios. As the baseline (0%), the figure considers the anticipated product quantity (540 tonnes per annum), which has been calculated with the assumptions listed in Table 3., and based on the bioprocess sequence from Flowsheet A (Fig. 10). The light blue marks the best-case scenario, that could be achieved with short-to-medium-term process optimization, and the dark blue marks the predicted worst-case scenarios, where Table 3. values would not be achieved. For the value of upstream reaction titer, the worst-case scenario is 1 g/L, while the best-case scenario is 23 g/L. For the cumulative step yield, the former case is based on a sequence of 4 process steps, each of 80% yield, while the latter is based on the same number of steps, where each enables achieving 95% yield

The recombinant indigo biosynthesis is a moderately investigated research topic. Several important achievements have been accomplished, although process optimization has not been thoroughly addressed by currently published literature. It is likely that the currently reported process attributes could be improved, even relatively quickly, if more activity would be devoted for research and experiments. On the other hand, as there is no record of an established or pilot large-scale bioprocess for indigo biosynthesis, it is an eventuality that the performance of the proposed process may be worse than the recorded small-scale values. Therefore, to assess how impactful process attributes are for the indigo biosynthesis output, a sensitivity analysis was performed (Fig. 12).

Biological aspects and bioprocess performance, including DSP, are more difficult to optimize and cannot be easily influenced. Their betterment would come from improvement in biological methods and engineering parameters, for example, from optimization of the host *E. coli* strain for indigo synthesis or increasing the DSP yields via understanding more about the system of *E. coli*—indigo product. In comparison, with available financial investment, factors, such as annual batch scheduling, plant size, fermenter capacity or other operating factors, are easier to manipulate to increase production. They may be modified in a more predictable manner, and thus have not been considered here.

The sensitivity analysis considers the percentage change in the indigo output for currently predicted medium-term best-case scenarios for optimized process values, and for worst-cases for the situation, when predicted Table 3. values would not be achieved on a large scale. As briefly mentioned, it is challenging to successfully scale-up bioprocesses performance, due to aspects, such as more difficult mixing or heat and nutrients dispersion (Cossar 2019). The first considered factor has been the titer of the key upstream reaction, which is the indole oxidation to indoxyl, as explained in “Upstream processing (USP) considerations” section. The second has been the cumulative yield of total bioprocess, which, based on Flowsheet 1. (Fig. 10), has been derived by multiplication of yields for a four-step sequence.

It can be concluded that perhaps substantial resources should be directed especially toward strain and reaction engineering, as the indigo output appears to be potentially much more sensitive to changes in the upstream reaction titer. The changes in the value of this factor result in both, the largest increase (+ 27.8%) and drop (- 94.4%) in the quantity supplied by the recombinant platform. It is worth mentioning, that the given maximal increase in Fig. 12, which is dictated by this factor, may not be the highest possible one in the long-term view. The best-case of 23 g/L has been chosen due to being stated as likely achievable by Berry et al. (2002), although no study directed to obtain such output has been found in the literature. The worst-case titer value of 1 g/L has been assumed, as many indigo-relate studies, that have been reported in the last years consider yields around that level as good, for example, if FMOs are employed (“Upstream processing (USP) considerations” section). However, it has been described earlier, that most recent studies employ microbial strains that have not been modified toward a high-flux tryptophan pathway, which may easily yield 18 g/L (Berry et al. 2002). This is likely as recent research focused on the assessment of the best biocatalyst for the key indigo pathway reaction and it is known that the mentioned modification has been made. The output that is 94.4% smaller, than the one calculated with Table 3. values, is, therefore, rather unlikely. However, it visualizes the influence of the presence or lack of the tryptophan pathway modification and the importance of strain engineering of indigo biosynthesis.

In comparison, the anticipated output fluctuations due to different yields from step yields could cause half the size decrease of biological platform production decrease (− 45.4%), and around one-third of the increase in the best-case scenario (+ 8.6%), when compared to the case of improved reaction titer. The process yield is for the total bioprocess, and has been derived from the multifaction of 4 predicted purification steps of Flowsheet A, including DSP of centrifugation, filtration, and formulation, for which the single-step yield usually oscillates in the range of 80–95% (Marichal- Gallardo and Alvarez 2012). Thus, the worst-case scenario has been assumed as the case, where the biological platform consists of 4 steps, where for each one, the step yield is 80%, while for the best-case scenario that value is 95% for each. If the bioprocess relies on 4 steps, and each has an efficiency of 95% yield, the total yield would be 81.5%, while if each would have 80% efficiency this value would drop to 41%.

#### Further considerations—how would the process optimization influence platform output? Would industrially relevant fermentation be achievable in future?

**Fig. 13:**
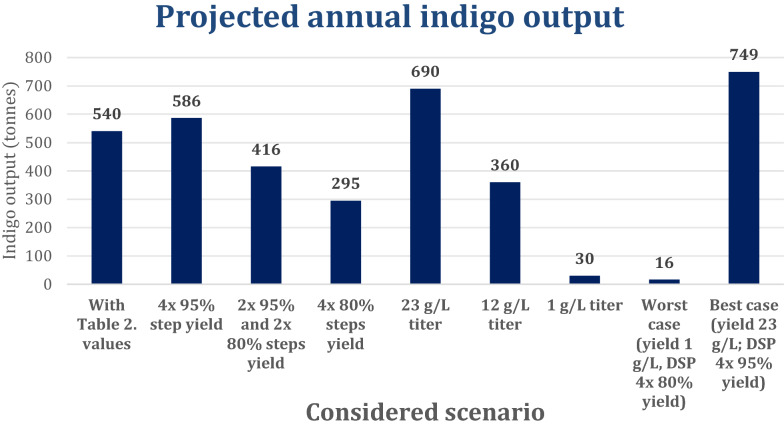
A graph that represents the projected annual output for a recombinant biological indigo platform depending on the alterations of 2 main values, that have been dictated by the Sensitivity Analysis presented in Fig. 12. The assumption is that platform relies on the bioprocess sequence from Flowsheet A (Fig. 10). In Fig. 12, the basic scenario has been fully based on Table 3. values, and equals 540 tonnes. Each graph represents a scenario, where a singular factor from Fig. 11 fluctuates—the reaction titer or the DSP step yields—or for their simultaneous alteration. The equation used for output calculations is the same as the equation that was used for Table 3. output estimation, which has been Output = (fermenter number) × (output from single fermenter per batch) × (batch runs/year) (Table 3.). The values used for calculations have been taken from Table 3. assumptions, unless stated otherwise. For example, the 2nd bar from the left was calculated with Table 3. inputs, except for DSP yield, which for calculation purposes has changed from 0.75% (top-down approach) total to 4 × 95% (bottom-up approach)

Figure 13 contains predicted outputs of recombinant indigo production, for several different scenarios that have been based on different possible cases dictated by the performed Sensitivity analysis (Fig. 12) Analysing these projections enables assessing the potential microbial fermentation potential and quantitatively visualizing what indigo outputs could be achieved in the short-to-medium-term period.

The output that was calculated earlier with Table 3. values, which is 540 tonnes, is considered as the basic case. Further graphs represent the feasible biological platform output in a case, where a singular factor Fig. 12 fluctuates—the reaction titre or the DSP step yields—or for their simultaneous alteration.

It has been described above that the currently relatively realistic scenario, which was based on the values reported in the literature, is already highly likely to be of interest to the indigo manufacturers, as the platform could satisfy the current annual indigo demand by an industrial denim dyeing unit (Table 3.). However, the Fig. 13 visualizes that this prediction possesses certain risks, and the output of the proposed biological platform would be especially sensitive to fluctuations in reaction titer. The basic scenario assumed that the indigo yields reported by Berry et al. (2002), achieved with the use of high-flux tryptophan pathway *E. coli* may be repeated, although in literature no later study that repeated such experiment is available. If a regular *E. coli* strain, that does not possess this modification would be harnessed, based on published small-scale experimental data, the yields could oscillate around 1 g/L at most (“Upstream processing (USP) considerations” section). Moreover, values nearly 1 g/L have been only reported so far for one class of potential biocatalysts, FMOs, while yields of other enzymes reach rather ~ 0.3 mg. The potential resulting radical decrease of output from 540 to 30 tonnes, or 16 tonnes when combined with poor DSP performance, would almost fully remove the attractiveness of the platform for industrial suppliers. Still, this is not a very probable scenario—strain engineering and modification is a well-developed and common mean in industrial biotechnology (Bailey et al. 1996; Olsson et al. 2022). It is widely used for the improvement of reaction performance, especially with the availability of an analytical tool for performing relevant strain screening in a high-throughput or automated manner (Marcellin and Nielsen 2018). Therefore, as some strain modifications have been reported in the literature, it is unlikely that they would not be harnessed or investigated in more detail before the employment of a certain bioprocess on an industrial scale.

The USP and DSP performance could influence the platform output, although the effect would not be as profound and critical as that of the reaction titre. If each of the process steps would achieve lower values of the usual range, this could reduce the output from 540 to 295 tonnes, which is substantially nearly ½ less. Still, rather than an extreme case of poor performance of all steps, a more probable intermediate scenario has been included, where 2 steps achieve high-end values and 2 achieve low-end yields. Such a situation is more probable, although the output loss of 416 tonnes, which is a 23% drop should not impede the potential of platform valuation significantly. A slightly stronger impact could be caused by the 2nd intermediate scenario, where the titre falls to 12 g/L, what causes produced dye quantity to fall by 1/3 to 360 tonnes.

In the context of potential improvement of considered process attributes over time, the optimization of process steps could increase indigo output to 586 tonnes. Although positive, this fluctuation is rather incremental than substantial and should not influence much the attractiveness of the platform any more to the potential stakeholders. In comparison, a further increase in reaction titre from 18 to 23 g/L, which was deemed possible by Berry et al. (2002), could result in achieving 690 annual indigo dye output, which is a significant increase of little less than 1/3. Moreover, in a long-term view, with simultaneous optimization of titre and process performance, the output could rise from 504 to 749 tonnes, representing a nearly 40% production increase. Therefore, with suitable capital investment and financing for such research activities, the platform has then the potential to supply indigo dye to more than multiple industrial dyeing units.

#### Process economics estimations—comparison of chemical and biological platforms

The commercial attractiveness of indigo biosynthesis to investors and business entities will be influenced by how its process economics compares to that of the currently prevalent chemical platforms. This paragraph aims to introduce the initial order-of-magnitude estimations, that are commonly included in the early stage considerations of individual engineering projects. For more accurate and thorough economic analysis engineers often employ specific software packages with tunable interfaces and modelling parameters, such as SuperPro Designer™ ( Petrides et al. 2019; Nandi et al. 2016) or Aspen Capital Cost Estimatior™ (Humbird 2021). As the assessed case scenario moves through conceptual, preliminary, and detailed design stages, aspects such as equipment sizing or facility location are narrowed down ( Ögmundarson et al. 2020, Harrison et al. 2015).

The technoeconomic projections generally categorize the required resources as related to Capital Investment (FCI) or Cost of Goods (known also as Operating costs). The fixed facility-related costs such as maintenance, taxation and labour are strongly related to the geographical area and facility lifetime. For indigo manufacturing, likely both, the biological and chemical plants would be set in a low-income country due to better financial parameters and the establishment of indigo suppliers in such regions (Paul et al. 2021). As discussed in “Overview of technically feasible bioprocesses and their contextual evaluation” section, indigo biosynthesis resembles low-value biological commodities and would have similar purity and quality requirements. Thus, Table 4. presents several examples of the capital investment valuation for such products. When benchmarked against these data, the potential capital investment for indigo biosynthesis should not exceed 300 million $. The outputs for presented common low-value bioproducts’ are significantly higher than the 540 tonnes estimated in this work for indigo biosynthesis. However, the yields for low-value commodities often reach 40 g/L (Rywińska and Rymowicz 2010), which is much higher, more than 2 × times, than 18 g/L assumed in this work for indigo (Berry et al. 2002). Thus, in a facility of the same size, the bioprocesses toward these other products are expected to yield much higher outputs, than a bioindigo platform.

**Table 4:** Exemplary low-value biological commodities with their respective capital investment values, fermenter sizes and product outputs

Commodity	Capital Investment (M$)	Product output (tonnes/year)	Fermenter size (m^3^)	References
Bioethanol	19–102	2800–24,000	N/A	Tsagkari et al. (2020)
Cultured meat	328	6800	480	Humbird (2021)
Microbial palm oil replacement - Case 1	16	8000	1000	Karamerou et al. (2021)
Microbial palm oil replacement - Case 2	23.5	16,000	2 × 1000	Karamerou et al. (2021)

To a certain extent, projections for chemical and biochemical plants often rely on data from similar projects or literature studies. The required engineering equipment is often similar across process engineering projects, such as extractors, centrifuges, or filters, as well as many direct utilities, such as water, energy, steam, or electricity (Harrison et al. 2015). For biopharmaceutical plants, equipment is often projected as more expensive, as the values 4–7 Lang values are often used instead of 3–5 common for chemical facilities (Farid et al. 2008).

This would not necessarily be the case for indigo, as biopharmaceuticals are rather high-value commodities (“Overview of technically feasible bioprocesses and their contextual evaluation” section). From the flowsheets presented in Fig. 10, the main proposed bioprocess, Flowsheet A, has a one-step longer purification sequence than Fig. 10D, which represented the standard manufacturing sequence for a synthetic dye. This could point to a slightly increased cost of biological manufacturing when compared to chemical manufacturing, although a direct comparison is difficult. This would require more detailed data on the process yields, precise material inputs during the multi-step chemical reactions, and the needed working and handling volume capacities for each platform. Figure 10 suggests that DSP of 10A and 10D is not drastically different and exploits similar product characteristics, such as solubility in solvents or particle size. Thus, if the processes’ scales would be similar, many of the required consumables such as filters, buffers, and the above-mentioned utilities would be similar. Moreover, while the waste treatment costs could theoretically very negatively impact the feasibility of chemical platforms, the relevant regulatory framework has not been implemented in developing countries yet. Thus, the potential taxation and waste management fees should not be accounted for yet (“Toxicity issues” section). Thus, major differences in operating costs should stem from the components required for the indigo synthesis reaction itself, the raw materials, as this is the key differentiating these two evaluated platforms (Figs. 7, 10). “Upstream processing (USP) considerations” section presented the relative pricing of possible starting materials for indigo biosynthesis and when compared with the main substrate for synthetic indigo, aniline, the cheapest glucose is still 3 × more expensive than the aromatic chemical (Additional file 1: Excel Spreadsheet). Therefore, the key gap to address in future technoeconomic assessments is the comparison between the economics of the upstream indigo synthesis step between the biological and chemical platforms. Preferably, this could harness one of the above-mentioned specialistic softwares.

## Conclusions

The massive global scale and the toxicity of current commercial synthetic indigo production create a market niche and demand for more sustainable and friendly processes, which may be effectively offered by biological fermentation platforms. All possible chemical pathways for this dye use unrenewable petrochemicals, harsh, hazardous compounds, and mostly highly elevated temperatures—these aspects are absent in the proposed microbial fermentation bioprocesses, which additionally offer substantial water savings.

The use of biological entities for indigo synthesis is assessed as an area that is relatively well-established within scientific research on a small, experimental scale. A number of key relevant discoveries has been made and this available data enabled to effectively consider many aspects in this paper and elsewhere, such as possible biocatalysts, fermentation modes, purification operations or biological platform outputs.

The currently projected process values and yields create an interesting commercial opportunity for indigo biosynthesis, that would be able to provide the dye amount required by a single industrial denim dyeing unit, with recognised space for further process optimisation in future. A small number of assumptions was made to facilitate the analysis, as commercial values are hard to find and not always publicly available. Thus, Figs. 9, 11, 12, and Table 3 are approximate. It is important for future reports to perform financial projections for the indigo synthesis step for biological and chemical indigo manufacturing. This would reliably assess the differences between these 2 platforms, and aid further optimisation of more sustainable pathways.

Nonetheless, the extent of the reported development of relevant research should effectively enable medium- to pilot-scale production ventures in the not-far future, when considering the global-wide shifts toward more sustainable processes and industry.

## Supplementary Information

**Additional file 1.** Publication: Production of indigo by recombinant bacteria. Additional file 1 Excel Spreadsheet 1. Mathematical Calculations.

## Data Availability

Data sharing is not applicable.

## Abbreviations

CE: Centrifugal extractor
COG: Cost of goods
CYP/P450: Cytochrome P450 monooxygenase
Da: Dalton (unit)
DNA: Deoxyribonucleic acid
DSP: Downstream processing
*E. coli*: *Escherichia coli*
FAD: Flavin adenine dinucleotide
FLPDA: Flavin-polydopamine
FMO: Flavin-dependent monooxygenase
h: Hour
L: Litre
LCA: Life cycle analysis or Life cycle assessment
LLE: Liquid–liquid extraction
mPh: Multicomponent phenol hydroxylase
min: Minute
NADH: Nicotinamide adenine dinucleotide
NADPH: Nicotinamide adenine dinucleotide phosphate
NDO: Naphthalene dioxygenase
*P. putida*: *Pseudomonas putida*
T: Temperature
UF: Ultrafiltration
UPO: Unspecific peroxygenase
USP: Upstream processing
